# Annexin A8 deficiency delays atherosclerosis progression

**DOI:** 10.1002/ctm2.70176

**Published:** 2025-01-21

**Authors:** Carmen Gutiérrez‐Muñoz, Rafael Blázquez‐Serra, Irene San Sebastian‐Jaraba, Sandra Sanz‐Andrea, Maria J. Fernández‐Gómez, Gonzalo Nuñez‐Moreno, Pablo Mínguez, Joan Carles Escolá‐Gil, Paula Nogales, Veronique Ollivier, Jose L. Martín‐Ventura, Benoit Ho‐Tin Noe, Ursula Rescher, Nerea Méndez‐Barbero, Luis M. Blanco‐Colio

**Affiliations:** ^1^ Vascular Research Laboratory, IIS‐Fundación Jiménez Díaz Madrid Spain; ^2^ CIBERCV, ISCIII Madrid Spain; ^3^ Bioinformatics Unit Department of Genetics & Genomics IIS‐Fundación Jiménez Díaz Madrid Spain; ^4^ CIBERER, ISCIII Madrid Spain; ^5^ Institut d'Investigacions Biomèdiques (IIB) Sant Pau Barcelona Spain; ^6^ CIBERDEM, ISCIII Madrid Spain; ^7^ Centro Nacional de Investigaciones Cardiovasculares Madrid Spain; ^8^ Department of Clinical Medicine Aarhus University Aarhus Denmark; ^9^ Laboratory for Vascular Translation Science Inserm U1148, Paris Bichat Hospital Paris France; ^10^ Center for Molecular Biology of Inflammation Research Group Regulatory Mechanisms of Inflammation, Institute of Medical Biochemistry, University of Muenster Muenster Germany

**Keywords:** AnxA8, atherosclerosis, inflammation

## Abstract

**Background:**

Atherosclerosis is a chronic inflammatory disease characterized by the accumulation of lipids and leukocytes within the arterial wall. By studying the aortic transcriptome of atherosclerosis‐prone apolipoprotein E (*ApoE^−/−^)* mice, we aimed to identify novel players in the progression of atherosclerosis.

**Methods:**

RNA‐Seq analysis was performed on aortas from *ApoE^−/−^* and wild‐type mice. AnxA8 expression was assessed in human and mice atherosclerotic tissue and healthy aorta. *ApoE^−/−^* mice lacking systemic AnxA8 (*ApoE^−/−^AnxA8^−/−^*) were generated to assess the effect of AnxA8 deficiency on atherosclerosis. Bone marrow transplantation (BMT) was also performed to generate *ApoE^−/−^* lacking AnxA8 specifically in bone marrow‐derived cells. Endothelial‐specific AnxA8 silencing in vivo was performed in *ApoE^−/−^* mice. The functional role of AnxA8 was analysed in cultured murine cells.

**Results:**

RNA‐Seq unveiled *AnxA8* as one of the most significantly upregulated genes in atherosclerotic aortas of *ApoE^−/−^* compared to wild‐type mice. Moreover, AnxA8 was upregulated in human atherosclerotic plaques. Germline deletion of AnxA8 decreased the atherosclerotic burden, the size and volume of atherosclerotic plaques in the aortic root. Plaques of *ApoE^−/−^AnxA8^−/−^* were characterized by lower lipid and inflammatory content, smaller necrotic core, thicker fibrous cap and less apoptosis compared with those in *ApoE^−/−^AnxA8^+/+^*. BMT showed that hematopoietic AnxA8 deficiency had no effect on atherosclerotic progression. Oxidized low‐density lipoprotein (ox‐LDL) increased AnxA8 expression in murine aortic endothelial cells (MAECs). In vitro experiments revealed that *AnxA8* deficiency in MAECs suppressed P/E‐selectin and CD31 expression and secretion induced by ox‐LDL with a concomitant reduction in platelet and leukocyte adhesion. Intravital microscopy confirmed the reduction in leukocyte and platelet adhesion in *ApoE^−/−^AnxA8^−/−^* mice. Finally, endothelial‐specific silencing of AnxA8 decreased atherosclerosis progression.

**Conclusion:**

Our findings demonstrate that AnxA8 promotes the progression of atherosclerosis by modulating endothelial−leukocyte interactions. Interventions capable of reducing AnxA8 expression in endothelial cells may delay atherosclerotic plaque progression.

**Key points:**

- This study shows that AnxA8 is upregulated in aorta of atheroprone mice and in human atherosclerotic plaques.
- Germline AnxA8 deficiency reduces platelet and leukocyte recruitment to activated endothelium as well as atherosclerotic burden, plaque size, and macrophage accumulation in mice.
- AnxA8 regulates oxLDL‐induced adhesion molecules expression in aortic endothelial cells. Our data strongly suggest that AnxA8 promotes disease progression through regulation of adhesion and influx of immune cells to the intima.
- Endothelial specific silencing of AnxA8 reduced atherosclerosis progression.
- Therapeutic interventions to reduce AnxA8 expression may delay atherosclerosis progression.

## Graphical abstract

Our work demonstrates a pathogenic role of endothelial Annexin A8 in atherosclerosis. The upregulation of AnxA8 in both human and mice atherosclerotic plaques strongly suggest that AnxA8 promotes disease progression through regulation of adhesion molecules expression and influx of immune cells to the intima. Therapeutic interventions to reduce AnxA8 expression in endothelial cells may delay atherosclerosis progression.

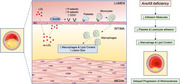

## 1 INTRODUCTION

Cardiovascular diseases (CVDs) are the leading cause of death in developed countries, accounting for nearly 18 million fatalities worldwide each year.^1^ The main underlying pathophysiology is atherosclerosis, a multifactorial inflammatory process involving several cell types, including endothelial cells (ECs), vascular smooth muscle cells (VSMCs) and leukocytes, which interact in response to various forms of injury.^2^ In an atherogenic environment, different stimuli such as hyperlipidaemia, hypertension or diabetes, induce ECs to express adhesion molecules that allow leukocytes to anchor to the intimal surface.^3^, ^4^ Subsequently, ECs and VSMCs begin to release proinflammatory cytokines, chemokines and vasoactive mediators to initiate and amplify the immune response leading to wall thickening. Monocytes are transformed into macrophages by internalization of oxidized low‐density lipoproteins (ox‐LDLs), and finally, macrophages differentiate into foam cells that contribute to lipid core formation. Platelets also contribute to atherosclerosis through their ability to form platelet‐neutrophil aggregates, which are mediated by adhesion molecules such as P‐selectin and P‐selectin glycoprotein ligand 1 (PSGL‐1).^5^ Platelet−neutrophil interaction leads to their reciprocal activation and neutrophil release of extracellular traps that are prothrombotic and mediate atherothrombosis.^6^ Persistent hypercholesterolemia and inflammation exacerbate atherosclerotic plaque progression over time, often complicated by thrombus formation, thereby leading to the possibility of developing an acute coronary syndrome or stroke.^7^ The identification of new molecules involved in vascular inflammation and atherogenesis could, therefore, help in the development of treatments to prevent plaque progression.

Annexins (Anxs) are a widespread multigene superfamily of structurally related calcium‐dependent membrane‐binding proteins.^8^ All annexins are structurally related, encoding a highly conserved C‐terminal core and a variable N‐terminal domain (also called the tail domain) containing sites for post‐translational modification, being unique in each member and responsible for conferring functional and regulatory properties to each annexin.^9^ Anxs are involved in numerous cellular processes including vesicle trafficking, calcium signalling, cell growth or division and apoptosis. Several mouse models lacking individual members of the Anxs gene family have been developed and some of their in vivo functions have been elucidated.^10^ In the context of CVD, recent studies have identified Anxs as proteins implicated in atherothrombosis.^11^ *AnxA1* deletion increased plaque size and leukocyte infiltration in atheroprone *ApoE^−/−^* mice.^12^ The N‐terminal portion of the AnxA1 peptide (i.e. Ac2‐26) is capable of reproducing the anti‐inflammatory effects of the full‐length protein. Thus, treatment with the Ac2‐26 peptide has been shown to reduce atherosclerotic lesion size and lesional macrophage accumulation in mice.^12^ Administration of AnxA5 reduced plaque inflammation in *ApoE^−/−^* mice probably by interfering with monocyte recruitment to the inflamed site.^13^ Targeting AnxA7 with 6‐amino‐2, 3‐dihydro‐3‐hydroxymethyl‐1, 4‐benzoxazine reduced atherosclerotic burden and leukocyte infiltration in *ApoE^−/−^* mice.^14^

AnxA8, like other members of the Anxs family, has been implicated in various steps of membrane trafficking. AnxA8 is located in late endosomes and is necessary for the delivery of CD63 to Weibel−Palade bodies (WPBs) and the presentation of P‐selectin on the surface of human ECs, which is crucial for leukocyte adhesion.^15^ Since inflammatory cell recruitment is a key event in atherosclerosis, here we investigated the importance of AnxA8 in atherogenesis and identified a prominent deleterious effect on plaque development and progression.

## 2 MATERIALS AND METHODS

### 2.1 Animals

Female *ApoE^−/−^* mice (#002052; Jackson Laboratory) were crossed with male *AnxA8^−/−^* mice previously generated in Dr. Rescher Lab.^15^ (both on the C57BL/6 background), and the progeny bred back to *ApoE^−/−^* mice to obtain the double knockout (*ApoE^−/−^AnxA8^−/−^*) and their littermate control (*ApoE^−/−^AnxA8^+/+^*) mice. To study the effect of *AnxA8* deletion on atherosclerotic lesions, 12‐week‐old male *ApoE^−/−^AnxA8^+/+^* (*N* = 8) and *ApoE^−/−^AnxA8^−/−^* (*N* = 8) were fed on a high‐fat diet (HFD; 21% fat [.2% cholesterol] +17.3% proteins; TD88137, Envigo) during 4 weeks (early lesions), and 24‐week‐old male *ApoE^−/−^AnxA8^+/+^* (*N* = 10) and *ApoE^−/−^AnxA8^−/−^* (*N* = 11) were fed on an HFD during 12 weeks (advanced lesions). Aorta from 40‐week‐old male *ApoE^−/−^* or wild‐type (WT) C57BL/6 mice (*N* = 4 for both) fed on a chow diet were used to obtain RNA for RNA‐Seq.

All mice were maintained under barrier conditions with water and diet available ad libitum. At the end of the study, 16‐h fasted mice were anesthetized and euthanized by overdose of 100 mg/kg ketamine and 15 mg/kg xylazine and saline perfused. Blood samples were collected and serum concentration of lipids was measured by using a commercial kit adapted for a COBAS 6000 autoanalyzer (Roche Diagnostics). The housing and care of animals and all procedures carried out in this study were strictly in accordance with the Directive 2010/63/EU of the European Parliament and were approved by the Institutional Animal Care and Use Committee of IIS‐Fundación Jiménez Díaz.

### 2.2 Bone marrow transplantation

Twelve‐week‐old male ApoE^−/−^ mice were irradiated with 4.5 Gy on 2 consecutive days to deplete the autologous bone marrow (BM). BM was collected from the femur and tibia of donor ApoE^−/−^AnxA8^+/+^ or ApoE^−/−^AnxA8^−/−^ mice by flushing with saline and erythrocytes were lysates. Each irradiated mouse was injected with 10^7^ BM cells through intravenous injection. Four weeks after bone marrow transplantation (BMT), peripheral blood was collected by retro‐orbital venous plexus puncture and the efficacy of BM reconstitution was tested by PCR analysis. For the atherosclerosis study, mice were fed with HFD for 12 weeks beginning 4 weeks after BMT. At the end of 12 weeks, mice were sacrificed and lipid analysis and atherosclerotic lesion analysis were performed.

### 2.3 RNA‐Seq library construction and sequencing

RNA‐Seq libraries were prepared using the Illumina TruSeq Stranded Total RNA library prep, after ribodepletion with the Epicenter Ribozero Gold kit (cat# RZE1224) starting from 500 ng of DNAse I treated total RNA, following the manufacturer's protocol, with the exception that 14 cycles of PCR were performed to amplify the libraries, to keep the duplication rate lower than with the recommended 15 cycles. The amplified libraries were purified using AMPure beads, quantified by Qubit and QPCR, and visualized in an Agilent Bioanalyzer. The libraries were pooled equimolarly, and loaded on an Illumina HiSeq 2500 flow cell, v4 chemistry as paired‐end 50. The R statistical software environment was used to run the Bioconductor package, DESeq2 to analyse the RNA‐Seq data set for differential expression between groups (Applied Bioinformatics Laboratory, NYU School of Medicine). Pairwise associations between up‐regulated genes in *ApoE* knockout (KO) compared to WT (FDR < .05) were extracted from the STRING database^16^ with a combined score of > 400. Markov Clustering algorithm clustering was performed using ClusterMaker cystoscape pluggin.^17^ Clusters with more than four genes were submitted to Gene Ontology (biological process) enrichment analysis implemented in Panther^18^ with mouse whole genome as background.

Datasets have been deposited in NCBIGene Expression Omnibus and are accessible through GEO series number GSE207414.

### 2.4 *En face* of aorta and aortic root morphometric analysis

Atherosclerotic lesions were quantified by *en face* analysis of the whole aorta and by cross‐sectional analysis of the aortic root. For *en face* preparations, the aorta was opened longitudinally, from the heart to the iliac arteries. After that, the aorta (from the heart to the iliac bifurcation) was ‘pinned out’ on a white wax surface in a dissecting pan using .2 mm stainless steel pins. After overnight fixation with 4% paraformaldehyde and a rinse in phosphate‐buffered saline (PBS), the aortas were immersed for 1 h in a filtered solution containing .2% Oil‐Red O (ORO) and unstained in 80% ethanol. The aortic arch was defined as the segment between the aortic valve and the descending aorta (3−4 mm behind the left subclavian artery). The thoracic aorta was defined as the segment between the descending aorta and the renal arteries. The ORO‐stained aortas were photographed and used for quantification of atherosclerotic lesions.

Hearts containing aortic roots were meticulously dissected and frozen in optimal cutting temperature compound. Aortic roots were sectioned at 5 µm thickness beginning proximally at the first evidence of the aortic valves at their attachment site of the aorta. From 0 to the end of the aortic valves, sections were stained with ORO/haematoxylin at 100 µm intervals. The volume of atherosclerotic lesions was estimated by calculating the area under the curve for each condition. The maximal lesion area was calculated for each mouse by averaging the values for three sections. The necrotic core was measured as a percentage of the total plaque area and was defined as a clear area that was Masson's trichrome stain‐free. Boundary lines were drawn around these regions, and the area measurements were obtained by image analysis software. Fibrous cap thickness was quantified by choosing the largest necrotic core from the sections of the aortic root of animals included in the advanced model and taking a measurement from the thinnest part of the cap, determined by measuring the distance between the outer edge of the cap and the necrotic core boundary.^19^

Picrosirius red staining was performed for analysis of collagen content by measuring birefringence to plane‐polarized light.

### 2.5 LDL retention in vivo

Fluorescently labelled human LDL was prepared as described.^20^ In brief, human blood from healthy donors was collected in K3E EDTA‐containing tubes (455036, Vacuette). After centrifugation, plasma was collected, mixed with KBr and layered on a KBr density gradient column. The column was centrifuged at 256 000*g* for 18 h in an Optima L‐100 ultracentrifuge (Beckman). The 1.063 g/mL density layer containing LDL was collected, purified on a PD10 column (17085101, GE Healthcare) and conjugated to the fluorochrome Atto 565 *N*‐hydroxysuccinimide ester (72464, Sigma‐Aldrich).

Mice received intravenous injections of Atto565‐LDL (15 µg/g body weight). Approximately 20 h postinjection, animals were anesthetized and euthanized by overdose of 100 mg/kg ketamine and 15 mg/kg xylazine and saline perfused. After overnight fixing in 4% formaldehyde/PBS, hearts were cut into 5 µm pieces and mounted in glycerol on a microscope slide. Fluorescence images were acquired with a Zeiss LSM780 confocal microscope. Autofluorescence (background) was determined in two mice (of each genotype) not injected with Atto565‐LDL. ImageJ Fiji software was used to quantify the percentage of the aortic area positive for red fluorescence.

### 2.6 Adeno‐associated in vivo transduction

Fifteen‐week‐old atheroprone *ApoE^−/−^* mice were given retro‐orbital injections of 1 × 10^11^ vector genome copies suspended in a total volume of 100 µL saline of pAAV2‐QuadYF‐Scramble‐EGFP (*N* = 8) or pAAV2‐QuadYF‐shAnxA8‐EGFP (*N* = 8) (Vectorbuilder). Target sequence AGGAGTGAGATTGACTTAAAT. After 1 week, mice were fed with HFD for 10 weeks. At the end of 10 weeks, mice were sacrificed and lipid analysis, transduced efficiency and atherosclerotic lesion analysis were performed.

### 2.7 Immunohistochemical analysis

Patients undergoing carotid endarterectomy who had carotid stenosis > 70% were included in the study for immunohistochemistry examinations. The area of the common carotid artery bifurcation was selected. Atherosclerotic plaques were collected at the time of the surgery. Informed consent was acquired prior to enrolment in all cases. In addition, healthy arteries were collected from deceased organ donors. Carotid samples were obtained with the authorization of the French Biomedicine Agency (PFS 09‐007, BBMRI network, BB‐0033‐00029). The study was performed in accordance with the principles outlined in the Declaration of Helsinki, and all participants gave written informed consent. A small portion of tissue from each sample was fixed in 3.7% paraformaldehyde for immunohistochemistry assessments. In addition, atherosclerotic plaques, as well as control carotid arteries, were placed in liquid N_2_ for later RNA extraction.

Immunohistochemical analysis was done as previously described.^21^ Primary Abs were the monocyte/macrophage marker CD68 (Ab53444; Abcam), and the smooth muscle cell marker smooth muscle actin (Clone 1A4; Sigma), endothelial marker CD31 (ab28364; Abcam), anti‐AnxA8 (Nbp3‐05654; NobusBio) and anti‐GFP (ab13970; Abcam). For human samples, anti‐AnxA8 (AF8105; R&D Systems) was used. Incubation without primary Abs and/or irrelevant species‐ and isotype‐matched immunoglobulins was used as a negative control for all immunostainings. Anti‐rat Alexa Fluor 488 and anti‐rabbit Alexa Fluor 488 or 543 were used as secondary Ab. For the detection of apoptosis, the TMR red‐labelled nucleotides (TUNEL) method was applied to the sections with an In Situ Cell Death Detection Kit TMR red according to the instruction (Roche Diagnostics). Image‐Pro Plus software was used to conduct computer‐assisted morphometric analysis (version 1.0 for Windows). The threshold setting for area measurement was equal for all images. Samples from each animal were examined in a blinded manner. Results were presented as the percentage of positive area versus total area (collagen, macrophages and α‐actin) or proportion of positive cells versus total cells (TUNEL).

### 2.8 Real‐time intravital imaging

Twelve‐week‐old *ApoE^−/−^AnxA8^+/+^* and *ApoE^−/−^AnxA8^−/−^* (*N* = 6 per group) were fed with HFD (4 weeks) to initiate atherosclerosis and induce recruitment of platelets and leukocytes to the carotid artery. Mice were anesthetized with ketamine/xylazine (100 and 10 mg/kg, respectively), injected with fluorescent tracers through the retro‐orbital venous sinus, their left and right common carotid arteries were exposed by surgical incision, and blood−cell interactions with the vessel wall were observed through an upright fluorescence macroscope (MacroFluo, Leica Microsystems) equipped with a thermostated heating plate and a 20X long distance objective, and connected to a sCMOS camera (Orca‐Flash‐4.0, Hamamatsu Photonics) for image acquisition using Metamorph software (Molecular Devices). Circulating platelets and leukocytes were stained with rhodamine‐6G (3 mg/kg mouse). Specific staining of platelets was achieved by injection of an Alexa 647‐conjugated rat monoclonal antibody to mouse GPIX (Emfret Analytics).

For the analysis of leukocyte interactions with the activated endothelium, mesenteric venules were exposed through a laparotomy and stimulated with calcium ionophore A23187 (10 µM) applied by superfusion, as described previously.^22^

Leukocyte rolling flux and the number of adherent leukocytes were determined by transillumination intravital microscopy and measured with ImageJ (National Institutes of Health). Cell adhesion was quantified by counting of adherent leukocytes in a 100 µm length. Rolling leukocyte was determined from the number of cells crossing a fixed line of venule per minute. Rolling velocity was calculated by measuring the distance covered by rolling cells divided by the time of rolling.

### 2.9 Cell culture

Murine aortic VSMCs were isolated from the aorta of *ApoE^−/−^AnxA8^+/+^* and *ApoE^−/−^AnxA8^−/−^* as previously described.^23^ Briefly, the aorta was carefully removed from the iliac arteries to the heart and immersed in PBS. Aortas were physically cleaned, cut into small pieces, and then enzymatically treated with 4 mg of collagenase per aorta (Sigma Life Science) in Dulbecco's minimum essential medium (DMEM)–F12 medium (Thermo Fisher Scientific) supplemented with 20% FBS. Then, cells were seeded in culture plaques and incubated for 5 days at 37°C. Murine aortic ECs (MAECs) were isolated from the aorta of *ApoE^−/−^AnxA8^+/+^* and *ApoE^−/−^AnxA8^−/^* ^−^ as previously described.^24^ Briefly, aortas were sectioned in small rings and incubated in DMEMF12 (Sigma) containing 1 mg/mL collagenase (type II, 290 U/mg), penicillin (100 U/mL), streptomycin (100 µg/mL) and glutamine (2 mmol/L) (Sigma) for 40 min at 37°C in 95% air/5% CO_2_. Then, the reaction was stopped with DMEM with 20% FBS, and cells were seeded in plastic culture flasks (Costar) previously coated with .5% sterile gelatin in DMEMF12 with 20% FBS, containing heparin (10 U/mL) and ECGF (100 U/mL). Cells were maintained in a humidified atmosphere of 5% CO_2_ in air at 37°C. After 5 days of incubation, primary ECs were selected from the culture by incubation with an anti‐mouse CD102 (BD Pharmigen 553326) antibody. Next, the secondary antibody associated with magnetic beads (Dynabeads anti‐rat IgG from CELLection) was incubated for 30 min with constant shaking. MAECs were seeded on plates previously treated with .5% gelatin. MAECs and VSMC were harvested for passaging at 2‐ to 3‐day intervals and used between the second and seventh passages.

For experiments using ox‐LDLs, LDLs from a pool of serum of healthy donors were obtained by ultracentrifugation and oxidized by incubation with 5 mM CuSO_4_ for 18 h at 37°C.

### 2.10 In vitro adhesion flow chamber assay

For cell adhesion experiments in flow conditions, MAECs from *ApoE^−/−^AnxA8^+/+^* and *ApoE^−/−^AnxA8^−/^* ^−^ aortas were seeded in perfusion chambers (Vena8 Endothelial+ biochips, Cellix) and grown under flow conditions (KIMA pump, Cellix) following the manufacturer instructions. Briefly, each microchannel of the biochip was coated overnight with 100 µg/mL of fibronectin (Promocell). After the coating period, cells were added in each microchannel and the biochip was placed at 37°C at 5% CO_2_ for 2 h before connection to the pump and then exposed to a continuous flow. After 24 h, MAECs were treated by flow perfusion of ox‐LDLs (100 µg/mL) in the medium for 18 h. The next day, blood from *ApoE^−/−^AnxA8^+/+^* or *ApoE^−/−^AnxA8^−/−^* animals was extracted by retro‐orbital capillarity to avoid platelet activation. Blood samples were incubated with a specific antibody to platelets (GPIX AF647). Subsequently, whole blood was perfused with an Exigo pump (Cellix) over the endothelium monolayer for 5 min at a venous shear stress of 2 dynes/cm^2^. Images were obtained from at least 10 different microscopic fields. The percentage of platelet adhesion was monitored and quantified by Image J.

### 2.11 In vitro static adhesion assay

MAECs from *ApoE^−/−^AnxA8^+/+^* and *ApoE^−/−^AnxA8^−/^* ^−^ were seeded and grown in a gelatin‐coated chamber slide (Lab‐Tek II) until confluency. Cells were starved for 18 h in FBS .5% serum before stimulation with ox‐LDL (100 µg/mL) for 4 h. Blood from *ApoE^−/−^AnxA8^+/+^* animals were drawn and peripheral blood mononuclear cells (PBMCs) were obtained by Histopaque gradient (1077/1119). PBMCs were counted and stained with Calcein (5 µM) for 30 min at room temperature (RT), and then washed twice. PBMCs (250 000 cells/well) were resuspended in DMEMF12 and added over the MAECs monolayer. After 20 min of incubation, the adhesion of PBMCs was evaluated by calcein expression over the endothelial monolayer (number of cells/per analysed field/10 field).

### 2.12 Lentiviral infection

For AnxA8 reconstitution in MAECs, lentiviruses expressing GFP (control) or mouse *Anxa8* mRNA were purchased from Applied Biological Materials. MAECs of *ApoE^−/−^AnxA8^−/−^* were infected at a multiplicity of infection (MOI) = 3 over 5 h. The medium was then replaced with fresh DMEMF12 supplemented with 10% FBS, and cells were cultured for 24 h, .2% serum‐starved for 24 h and stimulated with oxLDL (100 µg/mL) for 4 h. Then, PBMCs and adhesion protocol was carried out as previously described.

### 2.13 Cell immunofluorescence

MAECs were grown on gelatin‐coated coverslips, stimulated with oxLDL (100 µg/mL) for 4 h, fixed, permeabilized and stained with primary antibodies, anti‐CD31 (ab28364; Abcam), anti‐P‐selectin (550289; BD Pharmingen), anti‐E‐selectin (WC3190896; BD Pharmingen), and secondary antibodies Alexa Fluor 488. Then, nuclei were counterstained with DAPI, and images were acquired with a fluorescence microscope (Carl Zeiss). Mean fluorescence intensity was measured using Zeiss Zen imaging software.

### 2.14 Foam cell formation

Peritoneal macrophages were obtained from *ApoE*‐deficient or *ApoE/AnxA8*‐double deficient mice by peritoneal lavage 4 days after intraperitoneal injection of 3% (wt/vol) thioglycolate. Cells were cultured for 24 h in RPMI supplemented with 10% FBS, L‐glutamine and antibiotics and then stimulated for 24 h in a medium containing 1% FBS. Cells were then incubated with 10 µg/mL Dil‐ox‐LDL (Thermo Fisher Scientific) for different time points. Cells were fixed for 15 min in 4% paraformaldehyde and stained with DAPI. The percentage of foam cells was analysed in 5–10 randomly different fields per cell culture.

### 2.15 RT and real‐time PCR analysis

Human atherosclerotic tissues, aortic wall tissues from healthy human controls (.2 g for both) and total aorta from *ApoE* ^−/−^ or WT mice were snap‐frozen in liquid nitrogen, homogenates were resuspended in TRIzol buffer (Life Technologies) and total RNA was purified. Similarly, lysates from MAECs or VSMCs were resuspended in TRIzol, and total RNA was purified. Duplicate samples were quantified by determining absorbance at 260 nm. Real‐time PCR was performed on a TaqMan ABI 7700 Sequence Detection System using heat‐activated TaqDNA polymerase (Amplitaq Gold). After an initial hold of 2 min at 50°C and 10 min at 95°C, the samples were cycled 40 times at 95°C for 15 s and 60°C for 60 s. The expression of target genes was normalized to housekeeping transcript (18S/glyceraldehyde‐3‐phosphate dehydrogenase [*GADPH*]).

TaqMan probes *Vcam‐1* (Mm01320970_m1), *Icam‐1* (Mm00516023_m1), *Il‐6* (Mm00446190_m1), *Ccl2* (Mm00441242_m1) and Human *AnxA8* (Hs04190981) were purchased from Applied Biosystems and optimized according to the manufacturer's protocol. Mouse mRNA levels for *Anxa8, Cdkn1c, Pecam1, Selp, Sele, Acta2, Sm22, Klf4, Mmp9, Cd36, SRA, Abca1* and *Abcg1* were done by amplification of cDNA using SYBR Premix Ex TaqTM (Takara Biotechnology). The primer sequences are summarized in Table S1. All measurements were performed in triplicate. The 2ΔCT relative quantification method was used to assess the amount of target mRNA present in the samples. The values of each sample were calculated as multiples of their baseline values.

### 2.16 Western blot and ELISA

Cultured MAECs from different experimental conditions were collected and lysed in lysis buffer containing 50 mM Tris‐HCl pH 7.4, 150 mM NaCl, 2 mM EDTA, 2 mM EGTA, .2% Triton X‐100, .3% NP‐40, .2 mM PMSF, .2 mM Na_3_VO_4_ and pelleted.

Cell lysates were resuspended in an sodium dodecyl sulfate (SDS) sample buffer after adjusting for equal protein concentration and then separated by SDS‐PAGE, as previously described.^23^ After the proteins were transferred onto nitrocellulose membranes, the following antibodies were used to probe the membranes: AnxA8 (NBP3‐05654; Novus), phospho‐Akt (Ser473; #9271, Cell Signaling), Akt (#9272; Cell Signaling) and alpha‐tubulin (T5168; Sigma). Soluble concentrations of the adhesion molecules P‐selectin, E‐selectin, CD31 or proinflammatory cytokines CCL2 and CCL5 were measured in the culture medium or in the serum of mice by ELISA following the manufacturer's instructions (EMSELP and EMICAM1ALPHA, Thermo Fisher Scientific; ab204527, Abcam; DY478 and DY479, R&D Systems; respectively).

### 2.17 Statistical analysis

Data are presented as mean ± standard error of the mean (SEM) and the statistical significance of the differences was evaluated with the Student's *t*‐test or ANOVA Tukey's post‐hoc for multiple comparisons. Significance was accepted at the level of *p* < .05. Data analysis was performed using GraphPad Prism 6.0a software (GraphPad).

## 3 RESULTS

### 3.1 AnxA8 expression is upregulated in human and mice atherosclerotic plaques

**FIGURE 1:**
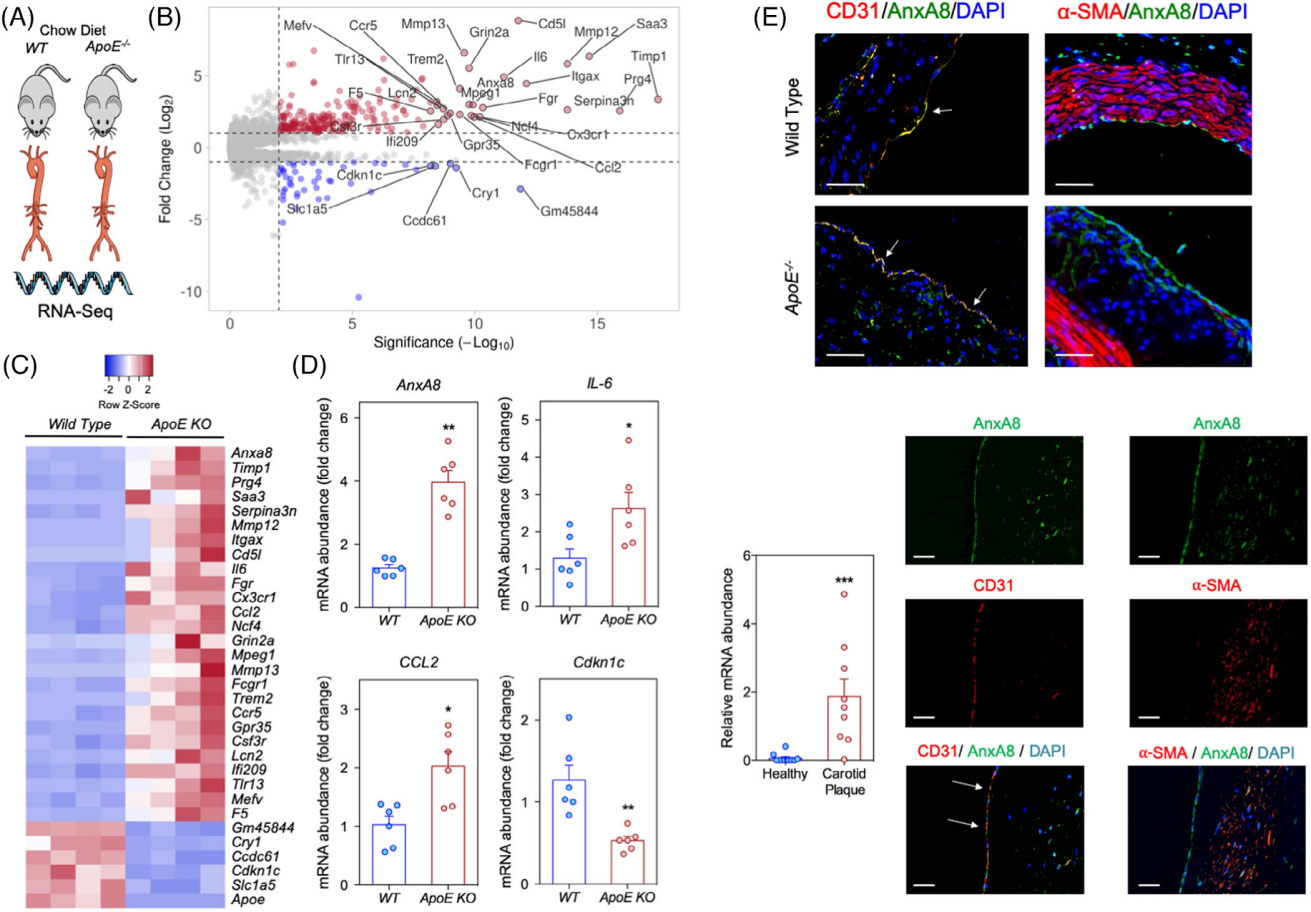
AnxA8 expression is upregulated in human and mice atherosclerotic plaques. (A) through (C), RNA‐Seq analysis of aortas from four *ApoE^−/−^* mice with four WT mice (40‐week‐old male mice). (B) Volcano plot showing the 15 643 expressed genes. Significantly differentially expressed genes are depicted in red (upregulated) or blue (downregulated) colour (‐adjusted *p‐*value < .01 and Log_2_ fold change > 1). The dashed lines indicate fold change and *p*‐value significance threshold. The names indicated belong to the 30 genes with the largest statistically significant difference. (C) Heat map representing of 30 genes with the largest statistically significant difference between wild‐type and *ApoE^−/−^* mice. *N* = 4 animals per group. (D) Validation by RT‐qPCR of selected genes identified by RNA‐Seq. Relative *AnxA8, CCL2, IL‐6* and *Cdkn1c* mRNA expression levels normalized to *GAPDH* mRNA expression of WT (*N* = 6) or *ApoE^−/−^* (*N* = 6) aortas. Data represent mean ± SEM. Student's *t*‐test. * *p* < .05 versus WT and ** *p* < .01 versus WT. (E) Immunostaining of AnxA8 (green) and its colocalization with ECs marker CD31 (red), and VSMCs marker α‐SMA (red) in aortic root sections of WT or *ApoE^−/−^* mice. Scale bar, 50 µm. (F) mRNA expression of *AnxA8* in human carotid atherosclerotic plaques (*N* = 9) or healthy aortas (*N* = 10) and immunofluorescence staining of CD31, AnxA8 and α‐SMA in human atherosclerotic plaques. Data represent mean ± SEM. Student's *t*‐test. *** *p* < .001 versus healthy. Scale bar, 50 µm.

To unveil genes that are differentially expressed in atherosclerosis, mRNAs from the aortas of 40‐week‐old atherosclerosis‐prone *ApoE* ^−/−^ mice (*N* = 4) were compared with aortas of sex‐ and age‐matched WT mice (*N* = 4) fed with chow diet to avoid spontaneous plaque formation. Reproducibility was high at the level of individual samples as evident from principal component analysis (Figure S1A). RNA‐Seq analysis revealed 743 genes differentially expressed in *ApoE* ^−/−^ mice, 483 up‐ and 260 down‐regulated (adjusted *p*‐value by FDR < .05; Figure S1B). As expected, gene ontology‐enriched analysis revealed T cell differentiation, the adaptive immune system and inflammatory response as the major pathways upregulated in the aorta of *ApoE*‐deficient versus WT mice (Figure S1C). Among the differentially regulated transcripts, we identified many genes that have been previously associated with atherosclerotic plaque development, including chemokines such as *Ccl2* and *Cx3cl1*, interleukins such as *IL‐6*, metalloproteinases or their inhibitors such as *Mmp12, Mmp13* and *Timp1*, or the proto‐oncogene *Fgr* (Figure 1A−C). Additionally, we also found several up‐ or down‐regulated genes whose role during atherosclerosis progression has not been previously characterized. To confirm the results obtained in RNA‐Seq, a panel of four genes differentially expressed in atherosclerotic aorta were independently validated by qRT‐PCR in a new set of aortas isolated from both *ApoE^−/−^* and WT mice. In line with the data obtained in RNA‐Seq, *AnxA8*, *IL‐6* and *Ccl2* were significantly up‐regulated and *Cdkn1c* was down‐regulated in aortas of *ApoE^−/−^* mice compared with those from WT mice (Figure 1D), with *AnxA8* being one of the most significantly up‐regulated genes in both RNA‐Seq and qRT‐PCR analysis (Figure 1A−D).

Immunohistochemistry analysis of the aortic sinus showed that AnxA8 colocalized with the EC marker CD31 in WT mice (Figure 1E). AnxA8 was expressed mainly in ECs in atherosclerotic plaques from *ApoE^−/−^* mice (Figure 1E and Figure S2). In line with the results obtained in the murine model, human carotid atherosclerotic plaques also showed significantly higher *AnxA8* mRNA expression compared with healthy arteries (Figure 1F), and AnxA8 co‐localized markedly with CD31 and α‐SMA in human atherosclerotic plaques (Figure 1F and Figure S2).

### 3.2 Germline *AnxA8* deficiency attenuates the progression of atherosclerosis

**FIGURE 2:**
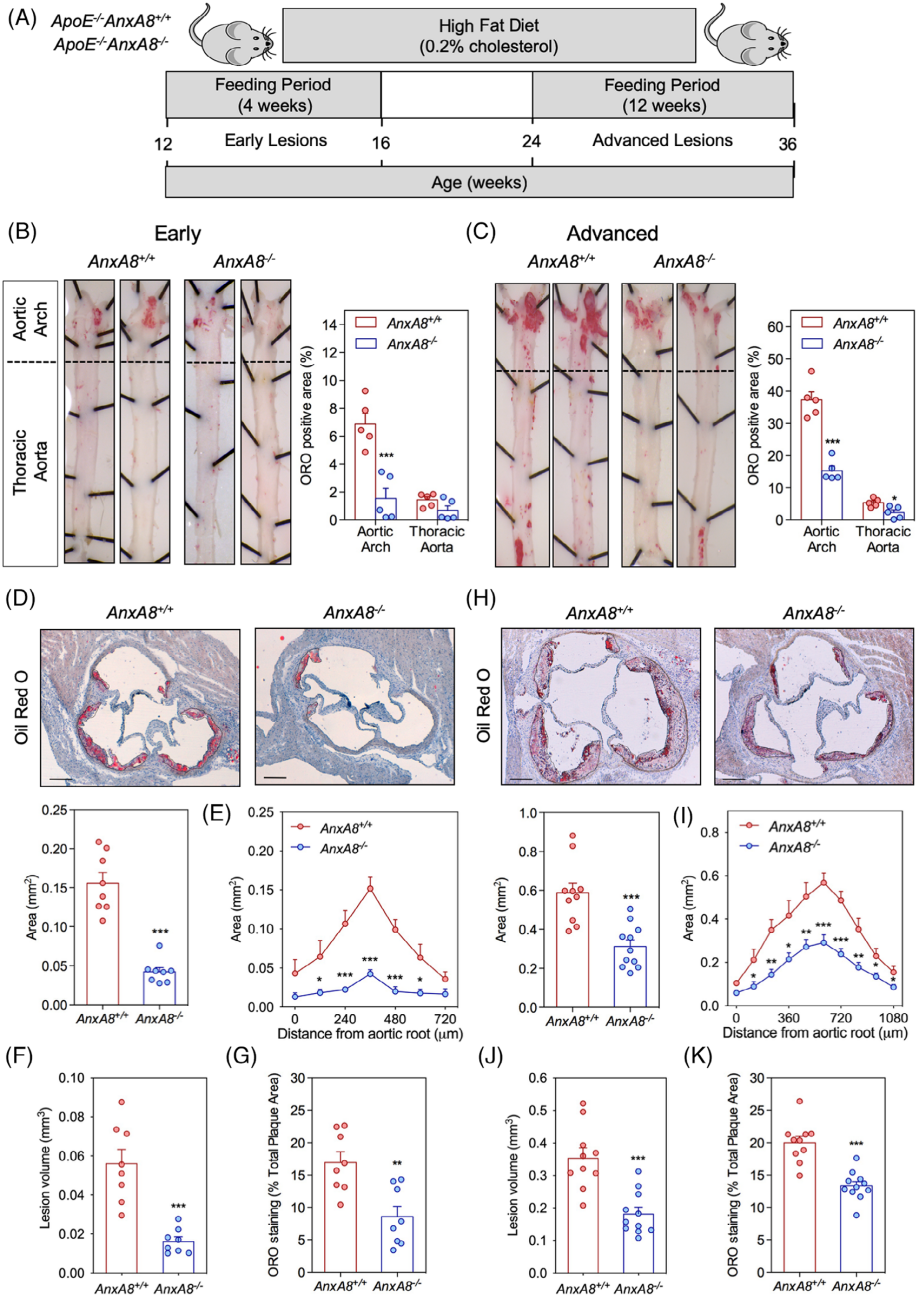
Germline *AnxA8* deficiency attenuates atherosclerosis progression. (A) Work flow for generation of early and advanced atherosclerotic lesions in *ApoE^−/−^AnxA8^+/+^* or *ApoE^−/−^AnxA8^−/^* ^−^ mice fed with a high‐fat diet. (B) Representative pinned‐out *en face* aorta preparations and quantification of atherosclerosis from 16 (early lesions) or (C) 36 (advanced lesions) weeks‐old mice stained with ORO. Data represent mean ± SEM of five animals per group. Student's *t*‐test. * *p* < .05 versus *ApoE^−/−^AnxA8^+/+^* and *** *p* < .001 versus *ApoE^−/−^AnxA8^+/+^*. (D) Representative ORO/haematoxylin staining and quantification of maximal lesion area in the aortic root of 16 (early lesions) weeks‐old mice. Data represent mean ± SEM of 8–11 animals per group. Student's *t*‐test. *** *p* < .001 versus *ApoE^−/−^AnxA8^+/+^*. Scale bars, 200 µm. (E) Quantification of atherosclerotic lesion area along aortic root in 16 (early lesions) weeks‐old mice. (F) The volume of atherosclerotic lesions was estimated by calculating the area under the curve for each condition. Data represent mean ± SEM of eight animals per group. Student's *t*‐test. * *p* < .05; *** *p* < .001 versus *ApoE^−/−^AnxA8^+/+^*. (G) Quantification of ORO in the aortic root from 16 weeks‐old *ApoE^−/−^AnxA8^+/+^* or *ApoE^−/−^AnxA8^−/−^* mice. Data represent mean ± SEM of eight animals per group. Student's *t*‐test. ** *p* < .01 versus *ApoE^−/−^AnxA8^+/+^*. (H) Representative ORO/haematoxylin staining and quantification of maximal lesion area in the aortic root of 36 (advanced lesions) weeks‐old mice. Data represent mean ± SEM of 10–11 animals per group. Student's *t*‐test. *** *p* < .001 versus *ApoE^−/−^AnxA8^+/+^*. Scale bars, 200 µm. (I) Quantification of atherosclerotic lesion area along aortic root in 36 (advanced lesions) weeks‐old mice. (J) The volume of atherosclerotic lesions was estimated by calculating the area under the curve for each condition. Data represent mean ± SEM of 10–11 animals per group. Student's *t*‐test. * *p* < .05; ** *p* < .01*** *p* < .001 versus *ApoE^−/−^AnxA8^+/+^*. (K) Quantification of ORO in the aortic root from 36 weeks‐old *ApoE^−/−^AnxA8^+/+^* or *ApoE^−/−^AnxA8^−/−^* mice. Data represent mean ± SEM of 10–11 animals per group. Student's *t*‐test. *** *p* < .001 versus *ApoE^−/−^AnxA8^+/+^*.

To analyse the contribution of AnxA8 in the progression of atherosclerosis, we bred *AnxA8^−/−^* mice with the atheroprone mouse model, *ApoE^−/−^*, to generate *ApoE^−/−^AnxA8^−/−^* mice. *ApoE^−/−^AnxA8^−/−^* animals and the corresponding *ApoE^−/−^* littermates were fed an HFD for 4 weeks (early lesions) or 12 weeks (advanced lesions) (Figure 2A). No differences were observed in body weight or serum cholesterol, triglycerides, HDL‐c or LDL‐c concentrations between the different groups (Figure S3). We assessed the atherosclerotic lesions in the entire aorta by *en face* staining with ORO to visualize lipid‐laden atherosclerotic plaques. In early lesions, we found a 78% reduction of the *en face* aortic arch lesion area in *ApoE^−/−^AnxA8^−/−^* mice compared to *AnxA8^+/+^* mice (Figure 2B). In addition, a marked reduction was observed in the atherosclerotic lesion size and volume at the aortic root *ApoE^−/−^AnxA8^−/−^* mice compared with *AnxA8^+/+^* mice (≈ 70% reduction for both; Figure 2D−F, respectively). The advanced model also displayed a significantly reduced *en face* lesion area in both the aortic arch and thoracic aorta (≈ 60% reduction for both; Figure 2C), and a reduced plaque size and volume (≈ 50% reduction for both; Figure 2H−J, respectively).

The composition of the lesion, rather than the size of the plaque, determines the likelihood of its rupture. Whereas collagen fibres stabilize atherosclerotic plaques, lipid depositions make plaques more prone to rupture.^25^ We observed a marked reduction in lipid deposition in the aortic wall of *ApoE^−/−^AnxA8^−/−^* mice compared with *ApoE^−/−^AnxA8^+/+^* mice in both the early (50% reduction; Figure 2D,G) and the advanced model (34% reduction; Figure 2H,K). Macrophage foam cell formation as a result of the excess of lipid deposition is a critical step in atherosclerotic plaque development.^26^ Since we have observed that AnxA8‐deficient mice showed atheroma plaques with lower lipids content, we next evaluated whether AnxA8 regulates macrophages cholesterol metabolism. The reduced lipid accumulation in *ApoE^−/−^AnxA8^−/−^* macrophages could be an outcome of either reduced uptake of LDL, via scavenger receptors SR‐A and CD36, or increased cholesterol efflux, through ATP‐binding cassette transporters ABCA1 and ABCG1.^27^, ^28^ To this end, mice peritoneal macrophages were incubated with ox‐LDL, TNF‐α or INF‐γ during 24 h. We observed no differences in *Cd36*, *SR‐A*, *Abca1* or *Abcg1* mRNA expression between *ApoE^−/−^AnxA8^+/+^* or *ApoE^−/−^AnxA8^−/^* ^−^ macrophages (Figure S4A). In addition, peritoneal macrophages were incubated with fluorescently labelled Dil‐ox‐LDL (10 µg/mL; 2–6 h) and assessed lipid content by fluorescence incorporation. We observed no differences in lipid accumulation between *ApoE^−/−^AnxA8^+/+^* or *ApoE^−/−^AnxA8^−/^* ^−^ macrophages (Figure S4B). These data indicate that AnxA8 may not be involved with lipid metabolism in macrophages.

**FIGURE 3:**
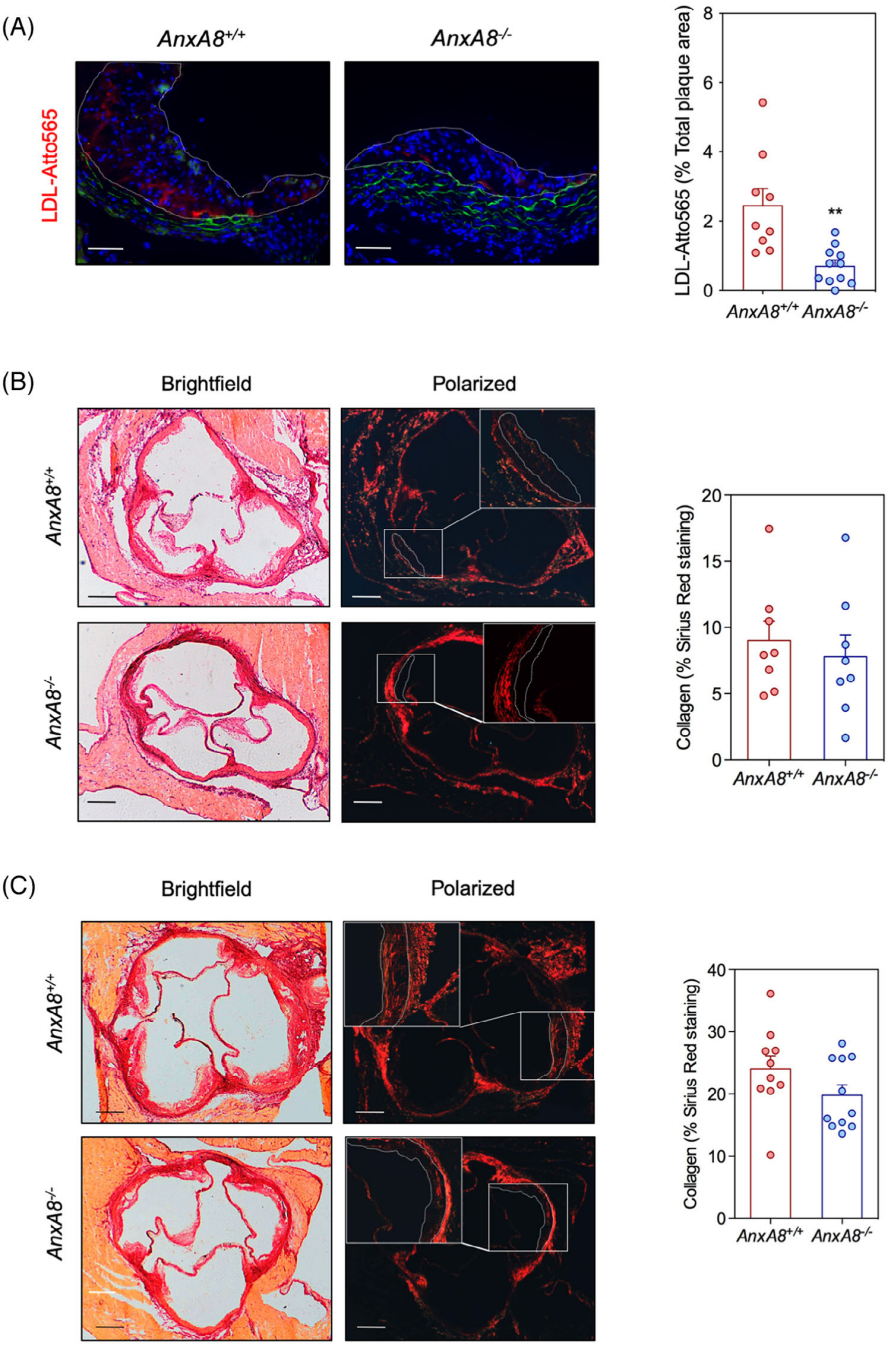
Germline *AnxA8* deficiency decreases LDL retention in atherosclerotic lesions. (A) Representative images and quantitative analysis of Atto565‐LDL retention in the aortic root of *ApoE^−/−^AnxA8^+/+^* or *ApoE^−/−^AnxA8^−/−^* mice after 20 h intravenous injection of LDL. Data represent mean ± SEM of 9–11 animals per group. Student's *t*‐test. ** *p* < .01. (B) Representative Sirius Red staining in the aortic root from 16 weeks‐old *ApoE^−/−^AnxA8^+/+^* or *ApoE^−/−^AnxA8^−/−^* mice. Quantification of the positive area is shown in the right panel. Scale bars, 200 µm. Data represent mean ± SEM of eight animals per group. (C) Representative Sirius Red staining in the aortic root from 36 weeks‐old *ApoE^−/−^AnxA8^+/+^* or *ApoE^−/−^AnxA8^−/−^* mice. Quantification of the positive area is shown in the right panel. Scale bars, 200 µm. Data represent mean ± SEM of 10–11 animals per group.

Since LDL retention in the arterial wall is one of the key processes in atherosclerosis progression, we performed in vivo LDL retention experiments to analyse whether AnxA8 deficiency affects lipoprotein retention. We examined the accumulation of fluorescently labelled exogenous human LDL within atherosclerotic plaques of the aortic root in intravenous injected *ApoE^−/−^AnxA8^+/+^* or *ApoE^−/−^AnxA8^−/−^* mice that had been fed an HFD for 1 week. Atto565‐LDL content was quantified 20 h postinjection, at which time exogenous human LDL is cleared from the circulation.^20^ Atherosclerotic lesions of *ApoE^−/−^AnxA8^−/^* ^−^ showed reduced LDL retention compared with *ApoE^−/−^AnxA8^+/+^* mice (Figure 3A).

**FIGURE 4:**
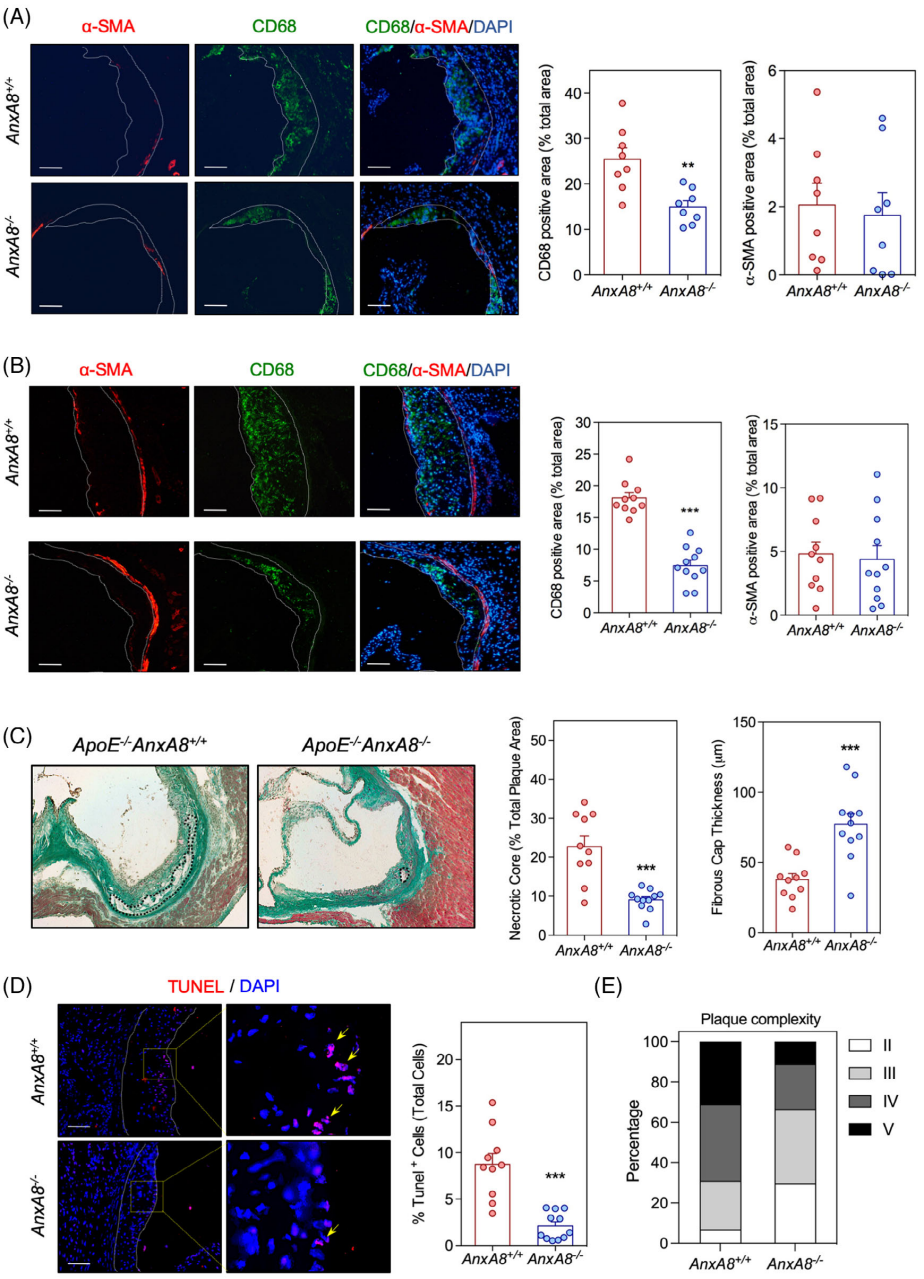
Germline *AnxA8* deficiency increases atherosclerotic plaque stability. (A) Representative histological analysis of cross‐sections of the aortic sinus from 16 (early lesions) or (B) 36 (advanced lesions) weeks‐old *ApoE^−/−^AnxA8^+/+^* and *ApoE^−/−^AnxA8^−/^* ^−^ mice stained with CD68, α‐SMA and DAPI. Quantification of macrophages or VSMCs content are shown in the panels below. Scale bars, 50 µm. Data represent mean ± SEM of 8–11 animals per group. Student's *t*‐test. ** *p* < .01 and *** *p* < .001 versus *ApoE^−/−^AnxA8^+/+^*. (C) Representative histological analysis of cross‐sections of the aortic sinus stained with Masson's trichrome from 36 weeks‐old *ApoE^−/−^AnxA8^+/+^* or *ApoE^−/−^AnxA8^−/^* ^−^ mice. Means of the necrotic core area and fibrous cap (FC) thickness calculated from Masson's trichrome–stained aortic cross‐sections were quantified and are shown (*N* *= *10 mice per group). Dashed lines indicate the boundary of the developing necrotic core (plaque acellular area). Scale bars, 100 µm. Student's *t*‐test. *** *p* < .001 versus *ApoE^−/−^AnxA8^+/+^*. (D) Representative histological analysis of cross‐sections of the aortic sinus stained with TUNEL. Quantification of TUNEL‐positive nuclei is shown in the graph on the right. DAPI was used to stain the nuclei. Scale bars, 50 µm. Data represent mean ± SEM of 10–11 animals per group. Student's *t*‐test. *** *p* < .001 versus *ApoE^−/−^AnxA8^+/+^*. (E) Histological classification of aortic root plaques complexity in the advanced model according to Stary's grading: II, early lesions (foam cells, SMCs, intracellular lipid accumulation); III, moderate lesions (foam cells, SMCs, pools of extracellular lipids); IV, atheroma (foam cells, SMCs, large pools of extracellular lipids, necrotic core); V, fibroatheroma (foam cells, SMCs, large pools of extracellular lipids, large irregular necrotic core).

We further characterized atherosclerotic plaques by analysing markers of inflammation and lesion stability. Collagen content was similar in atherosclerotic lesions present in the aortic root of both genotypes, in the early (Figure 3B) and in the advanced model (Figure 3C). Analysis of atheroma also revealed decreased accumulation of CD68^+^ cells, without affecting smooth muscle cell content in early and advanced lesions of *ApoE^−/−^AnxA8^−/−^* mice compared with *ApoE^−/−^AnxA8^+/+^* mice (Figure 4A,B; respectively). In addition, a significant increase in the thickness of the fibrous cap and a reduction in plaque necrosis were observed in advanced plaques from *ApoE^−/−^AnxA8^−/−^* mice compared with *ApoE^−/−^AnxA8^+/+^* mice (Figure 4C). Increased numbers of TUNEL‐positive cells were present in advanced lesions from *ApoE^−/−^AnxA8^−/−^* mice compared with *ApoE^−/−^AnxA8^+/+^* mice (Figure 4D). *AnxA8* ^−/−^ mice showed a decrease in overall plaque complexity, as measured by Stary scoring,^29^ with an increase in class II lesions compared to AnxA8^+/+^ mice, at the expense of class IV and V lesions (Figure 4E and Figure S5).

Since the phenotypic switch of vascular cells also plays an important role in atherosclerosis progression, we examined whether *AnxA8* deficiency influences VSMCs phenotype. mRNA expression of *Klf4* and *Mmp9*, synthetic VSMCs phenotype markers, were downregulated in the aorta of *ApoE^−/−^AnxA8^−/−^* mice compared with *ApoE^−/−^AnxA8^+/+^* mice (Figure S6A). By contrast, mRNA expression of *Acta2*, a VSMCs contractile marker, was upregulated in the aorta of *ApoE^−/−^AnxA8^−/−^* mice compared with *ApoE^−/−^AnxA8^+/+^* mice (Figure S6A).

To confirm that AnxA8 is involved in the phenotypic switch of VSMCs, cells were incubated in the presence of ox‐LDL or proinflammatory cytokines, and phenotype markers were analysed in a time‐dependent manner. TNF‐α, INF‐γ or ox‐LDL decreased *Acta2* mRNA expression in VSMCs (Figure S6B). *Mmp9* mRNA expression was also increased by ox‐LDL or TNF‐α. However, INF‐γ did not alter *Mmp9* mRNA expression in VSMCs. No differences were observed between VSMCs from *ApoE^−/−^AnxA8^−/−^* or *ApoE^−/−^AnxA8^+/+^* mice (Figure S6B). Our data may indicate that AnxA8 is not involved in the phenotypic switching of VSMCs and that the results observed in vivo may be related to the microenvironment within atherosclerotic plaques rather than to AnxA8 deficiency.

Finally, serum levels of proinflammatory cytokines CCL2 and CCL5 were reduced in *ApoE^−/−^AnxA8^−/−^* mice compared with *ApoE^−/−^AnxA8^+/+^* mice (Figure S7). Collectively, these results demonstrate that AnxA8 deficiency attenuates plaque progression, promotes plaque stability and reduces systemic inflammation in atheroprone mice.

### 3.3 *AnxA8* deficiency in hematopoietic cells does not affect atherosclerosis progression

**FIGURE 5:**
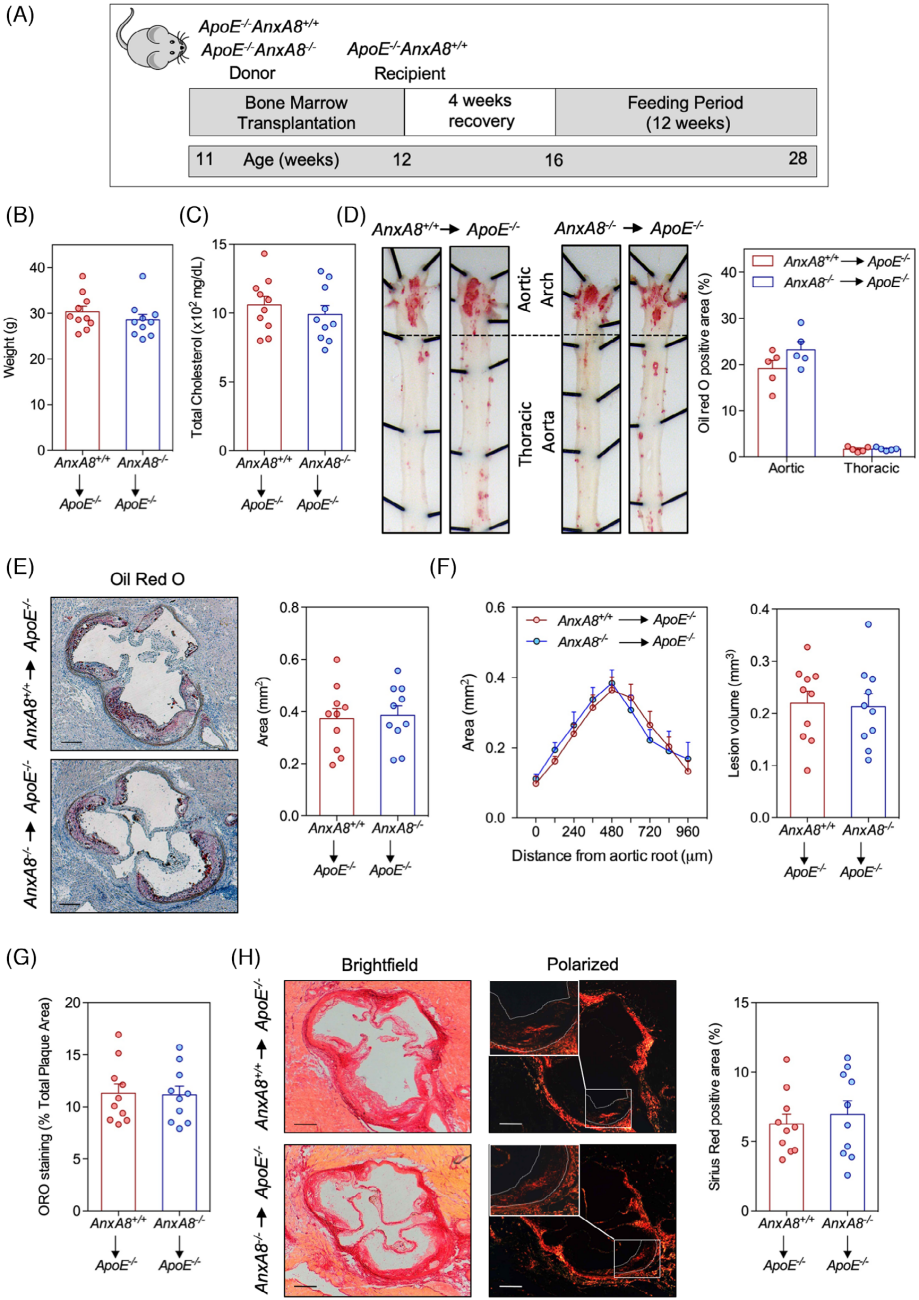
Hematopoietic *AnxA8* deficiency does not prevent atherosclerosis progression. (A) Work flow for generation of *ApoE^−/−^* chimeras with *AnxA8^+/+^* or *AnxA8^−/−^* BM. (B) Weight and (C) serum cholesterol concentrations of *ApoE^−/−^* chimeras with *AnxA8^+/+^* or *AnxA8^−/−^* BM after 12 weeks on HFD (mean ± SEM; *n* = 10 per group). (D) Representative pinned‐out *en face* aorta preparations and quantification of atherosclerosis from *ApoE^−/−^* chimeras with *AnxA8^+/+^* or *AnxA8^−/−^* BM stained with ORO after 12 weeks on HFD. Data represent mean ± SEM of five animals per group. (E) Representative ORO/haematoxylin staining and quantification of maximal lesion area in the aortic root of *ApoE^−/−^* chimeras with *AnxA8^+/+^* or *AnxA8^−/−^* BM after 12 weeks on HFD. Data represent mean ± SEM of 10 animals per group. Scale bars, 200 µm. (F) Quantification of atherosclerotic lesion area along aortic root in *ApoE^−/−^* chimeras with *AnxA8^+/+^* or *AnxA8^−/−^* BM after 12 weeks on HFD. The volume of atherosclerotic lesions was estimated by calculating the area under the curve for each condition. Data represent mean ± SEM of 10 animals per group. (G) Quantification of ORO in the aortic root from *ApoE^−/−^* chimeras with *AnxA8^+/+^* or *AnxA8^−/−^* BM after 12 weeks on HFD. Data represent mean ± SEM of 10 animals per group. (H) Representative Sirius Red staining in the aortic root from *ApoE^−/−^* chimeras with *AnxA8^+/+^* or *AnxA8^−/−^* BM after 12 weeks on HFD. Quantification of the positive area is shown in the right panel. Data represent mean ± SEM of 10 animals per group. Scale bars, 200 µm.

To analyse the contribution of AnxA8 deficiency in the hematopoietic compartment on atherosclerosis progression, *ApoE^−/−^* mice were lethally irradiated and transplanted with BM from *ApoE^−/−^AnxA8^−/^* ^−^ or *ApoE^−/−^AnxA8^+/+^* donor mice. After 1 month of BM reconstitution, mice were fed an HFD for 12 weeks (Figure 5A). No differences were observed in body weight or cholesterol concentrations between the different groups after transplantation (Figure 5B,C). When we analysed the atherosclerotic burden by *en face* in the aorta and lesion size or volume in the aortic root, no statistically significant differences could be found between *ApoE* ^−/−^ transplanted with hematopoietic cells from *ApoE^−/−^AnxA8^+/+^* or *ApoE^−/−^AnxA8^−/−^* mice (Figure 5D−F). Furthermore, ORO and collagen content (Figure 5G,H) as well as CD68 and α‐SMA positive area of the lesions (Figure S8) were similar between both groups of mice. Overall, these data indicate that leukocyte AnxA8 does not influence the progression of established lesions.

### 3.4 Endothelial AnxA8 regulates platelet adhesion in atheroprone mice

**FIGURE 6:**
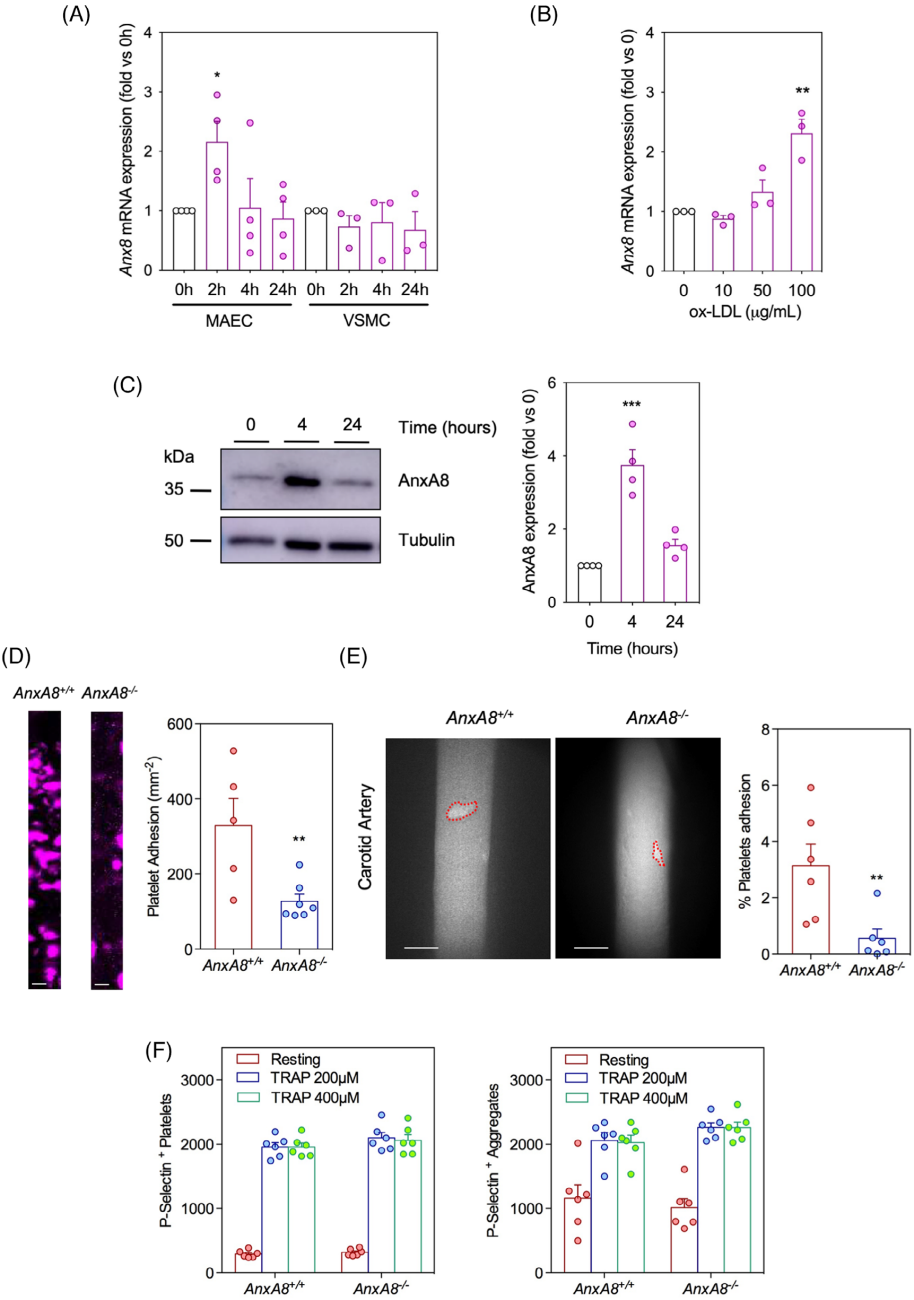
Expression of AnxA8 is increased by ox‐LDL in MAECs and platelet adhesion to endothelial cells in vitro and in vivo. (A) Relative *AnxA8* expression levels normalized to 18s rRNA of MAECs or VSMCs treated with or without ox‐LDL (100 µg/mL) for 0–24 h. Values shown are mean ± SEM of 3–4 different experiments. ANOVA Tukey's post hoc. * *p* < .05 versus 0 h. (B) Relative *AnxA8* expression levels normalized to 18s rRNA of MAECs treated with or without ox‐LDL (10–100 µg/mL) for 2 h. Values shown are mean ± SEM of three different experiments. ANOVA Tukey's post hoc. * *p* < .05 versus 0 h. (C) Representative western blot analysis of AnxA8 in MAECs treated with ox‐LDL (100 µg/mL) during 0−24 h. Tubulin was used as loading control. Values shown are mean ± SEM of four different experiments. ANOVA Tukey's post hoc. *** *p* < .001 versus 0 h. (D) Representative fluorescence images depicting platelets adhering to endothelial *ApoE^−/−^AnxA8^+/+^* or *ApoE^−/−^AnxA8^−/^* ^−^ surface for 5 min at a venous shear stress of 2 dynes/cm^2^ in a microfluidic flow condition. Data represent mean ± SEM of 5–7 different experiment per group. Student's *t*‐test. ** *p* < .01 versus *AnxA8^+/+^*. Scale bars, 200 µm. (E) Fluorescent intravital microscopy images illustrating the Alexa 647‐labelled platelets in incipient lesional carotids of *ApoE^−/−^AnxA8^+/+^* or *ApoE^−/−^AnxA8^−/−^* mice after 4 weeks of HFD. Quantification of percentage of platelets coverage area in the carotid zone analysed is represented in the right panel. Data represent mean ± SEM of six animals per group. Student's *t*‐test. ** *p* < .01 versus *ApoE^−/−^AnxA8^+/+^*. Scale bars, 200 µm. (F) Flow cytometry analysis of P‐selectin expression and platelets aggregation in *ApoE^−/−^AnxA8^+/+^* or *ApoE^−/−^AnxA8^−/−^* platelets. Bar graph representing the fluorescence intensity for P‐selectin of platelets and aggregates of non‐treated (resting) and TRAP‐stimulated *ApoE^−/−^AnxA8^+/+^* or *ApoE^−/−^AnxA8^−/−^* platelets.

Since our results indicated that AnxA8 deficiency in hematopoietic cells does not play a significant role in atherosclerosis, we next analysed AnxA8 expression in resident cells, specifically VSMCs and ECs. Treatment with ox‐LDL, the most common form of modified LDL in atherosclerotic plaques, increased AnxA8 mRNA and protein expression in MAECs (Figure 6A−C), but not in VSMCs (Figure 6A).

Platelet adhesion at atheroprone sites like the carotid arteries is one of the earliest steps in atherosclerosis.^30^ Therefore, we perfused whole blood from *ApoE* ^−/−^ *AnxA8^+/+^* mice over *AnxA8^+/+^* or *AnxA8^−/−^* MAECs prestimulated with ox‐LDL. A significantly higher number of platelets (detected with the fluorescent antibody against GPIX added to the blood) adhered to ox‐LDL stimulated WT ECs than to *AnxA8‐*deficient ECs (Figure 6D). A similar result was observed when whole blood from *ApoE* ^−/−^ *AnxA8^−/^* ^−^ mice was perfused over *AnxA8^+/+^* or *AnxA8^−/−^* MAECs prestimulated with ox‐LDL (Figure S9). We next assessed platelet interactions with the carotid arteries of *ApoE* ^−/−^ *AnxA8^+/+^* or *ApoE* ^−/−^ *AnxA8* ^−/−^ mice fed an HFD for 4 weeks. Significantly reduced spontaneous platelet adhesion was observed in carotid arteries of *ApoE* ^−/−^ *AnxA8* ^−/−^ compared to *ApoE* ^−/−^ *AnxA8^+/+^* mice (Figure 6E). Overall, our results suggest the role of AnxA8 as a regulator of endothelium−platelet interaction.

### 3.5 Endothelial AnxA8 regulates phenotypic features of cell activation

**FIGURE 7:**
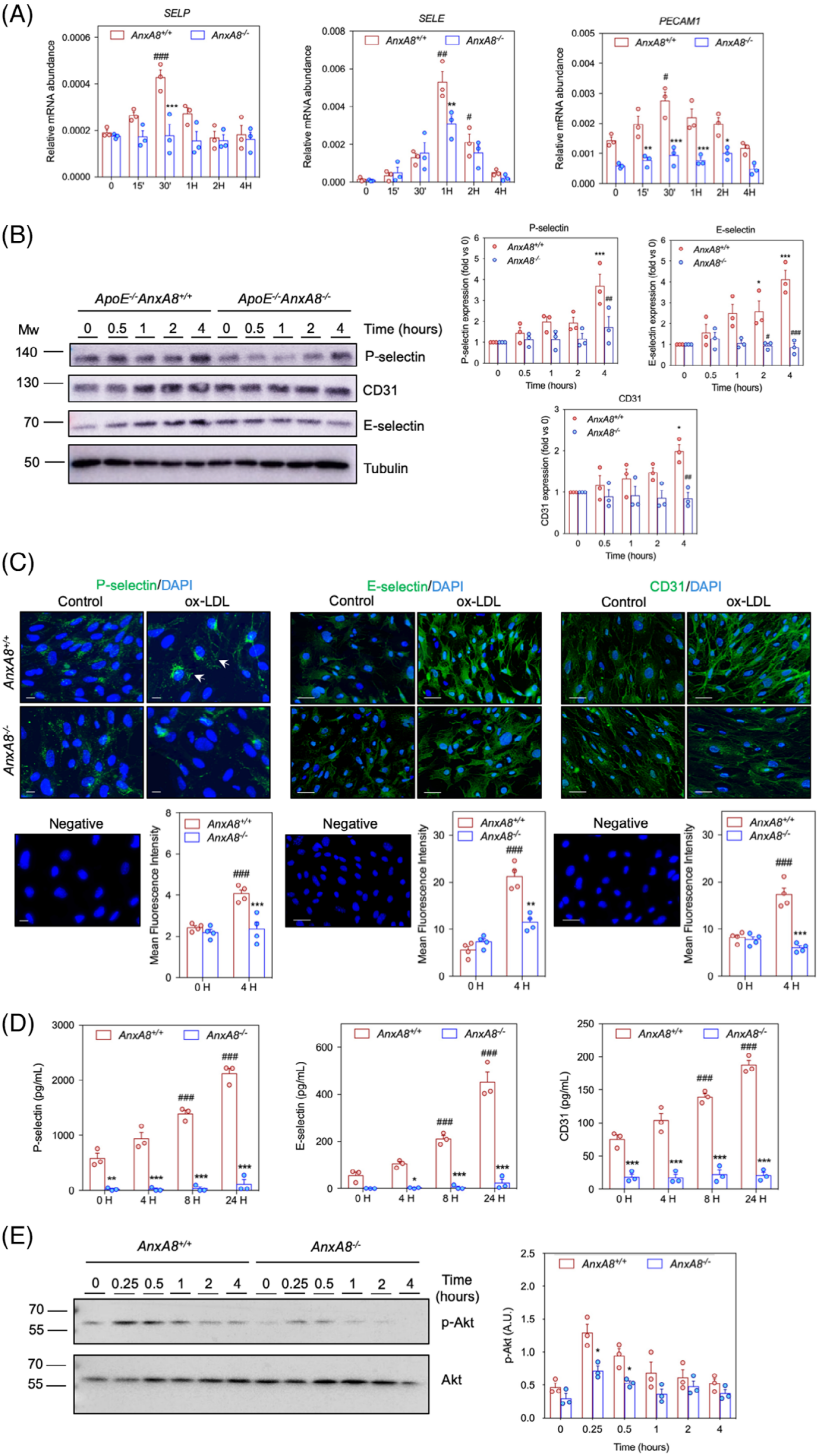
AnxA8 deletion diminishes adhesion molecules expression and secretion in aortic endothelial cells. (A) Relative *Selp, Sele* and *Pecam‐1* expression levels normalized to 18s rRNA of endothelial cells treated with or without ox‐LDL (100 µg/mL) for 0−4 h. The same cDNA was repeatedly tested for multiple genes. Values shown are mean ± SEM of three different experiments. ANOVA Tukey's post hoc. # *p* < .05 or ## *p* < .01 versus 0 h *ApoE^−/−^AnxA8^+/+^*; * *p* < .05, ** *p* < .01 or *** *p* < .001 versus *ApoE^−/−^AnxA8^+/+^*. (B) Representative western blot analysis of P/E‐selectin and CD31 in MAECs treated with ox‐LDL (100 µg/mL) during 0−4 h. Tubulin was used as loading control. Values shown are mean ± SEM of three different experiments. ANOVA Tukey's post hoc. *** *p* < .001 versus 0 h. (C) Representative immunofluoresce and mean fluorescence intensity of P‐selectin, E‐selectin or CD31 in endothelial cells treated with or without ox‐LDL (100 µg/mL) during 4 h. Scale bars, 50 µm. (D) Secretion of P‐selectin, E‐selectin or CD31 in endothelial cells treated with or without ox‐LDL (100 µg/mL) during 0−24 h. Values shown are mean ± SEM of three different experiments. ANOVA Tukey's post hoc. ### *p* < .001 versus 0 h *ApoE^−/−^AnxA8^+/+^*; * *p* < .05, ** *p* < .01 or *** *p* < .001 versus *ApoE^−/−^AnxA8^+/+^*. (E) Representative western blot analysis of p‐Akt and Akt in MAECs treated with ox‐LDL (100 µg/mL). Right panel shows the quantification of band densitometry values of p‐Akt protein levels expressed in arbitrary units after correction for Akt (loading control). * *p* < .05 or ** *p* < .01 versus 0 h. ANOVA Tukey's post hoc.

Increased expression of adhesion molecules by activated endothelium is a phenotypic feature in the first steps of atherosclerotic plaque initiation.^31^ To know whether changes in AnxA8 expression and localization could affect the activation of ECs under hyperlipidaemic conditions, primary MAECs from *ApoE* ^−/−^ *AnxA8^+/+^* or *ApoE* ^−/−^ *AnxA8* ^−/−^ were stimulated for different times with ox‐LDL. While ox‐LDL increases E/P‐selectin and Pecam‐1 mRNA and protein expression in *AnxA8^+/+^* MAECs, a limited effect was observed in *AnxA8*‐deficient MAECs (Figure 7A−C). In addition, P/E‐selectin and CD31 secretion to the supernatant was diminished in *AnxA8^−/−^* MAECs stimulated with ox‐LDL compared with control *AnxA8^+/+^* cells (Figure 7D). No significant differences were observed in VCAM‐1 or ICAM‐1 expression between *AnxA8^+/+^* or *AnxA8^−/−^* MAECs (Figure S10A).

Since platelets also express and store P‐selectin in α‐granules, we incubated blood samples from *AnxA8^+/+^* or *AnxA8^−/−^* mice in the presence or absence of thrombin receptor activator protein (TRAP). Flow cytometry analysis showed that, whereas unstimulated platelets did not express P‐selectin, incubation of blood of *ApoE* ^−/−^ *AnxA8^+/+^* or *ApoE* ^−/−^ *AnxA8^−/^* ^−^ mice with TRAP caused platelet activation and aggregation, with similar P‐selectin expression levels in platelets of both types of mice (Figure 6F and Figure S11).

As the Akt pathway has been shown to be involved in adhesion molecule expression in ECs,^32^, ^33^ we analysed whether AnxA8 might regulate Akt activation. We have observed that ox‐LDL increased Akt phosphorylation in MAECs, an effect that was partially prevented in AnxA8‐deficient MAECs (Figure 7E).

### 3.6 AnxA8 deficiency reduces in vivo leukocyte recruitment in atheroprone mice

**FIGURE 8:**
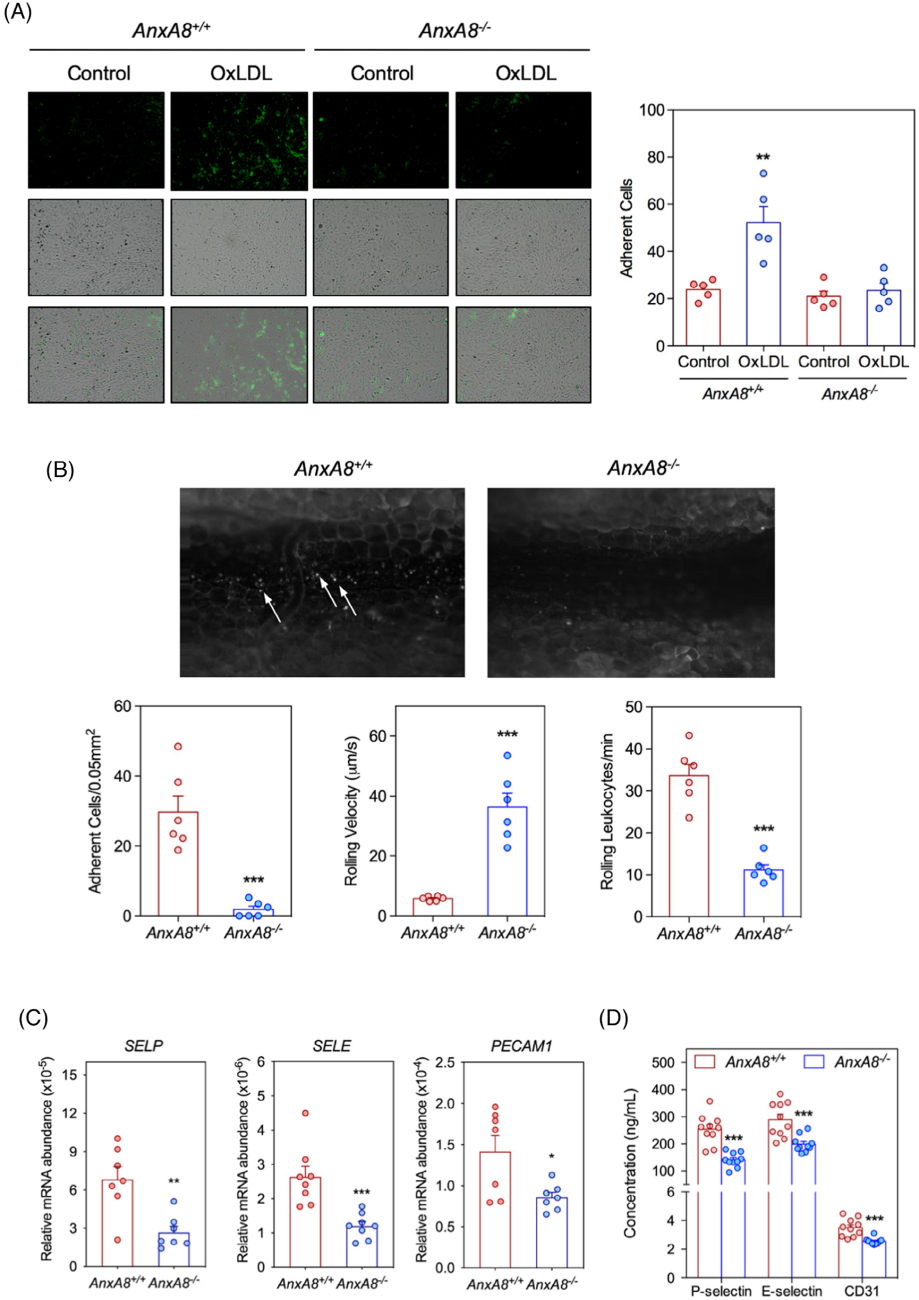
AnxA8 deficiency diminishes leukocytes adhesion to endothelial cells. (A) Representative fluorescence and light‐field images of calcein‐labelled PBMCs on *ApoE^−/−^AnxA8^+/+^* or *ApoE^−/−^AnxA8^−/−^* endothelial monolayer in the absence or presence of ox‐LDL. Quantification of the number of calcein‐labelled PBMCs in 10 random fields analysed in each condition. Values shown are mean ± SEM of five different experiments. Student's *t*‐test. ** *p* < .01 versus control. Scale bars, 100 µm. (B) Fluorescent intravital microscopy images of rhodamine 6G‐labelled leukocytes on calcium ionophore‐activated mesenteric venules of *ApoE^−/−^AnxA8^+/+^* or *ApoE^−/−^AnxA8^−/−^* mice after 4 weeks of HFD. Arrows indicated adhering leukocytes. Number of firmly adherent leukocytes, rolling velocity and rolling leukocytes are represented below. Data represent mean ± SEM of six animals per group. Student's *t*‐test. *** *p* < .001 versus *ApoE^−/−^AnxA8^+/+^*. Scale bars, 100 µm. (C) Relative *Selp, Sele* and *Pecam‐1* expression levels normalized to 18s rRNA of aorta from *ApoE^−/−^AnxA8^+/+^* or *ApoE^−/−^AnxA8^−/−^* mice. The same cDNA was repeatedly tested for multiple genes. Values shown are mean ± SEM of seven animals per group. Student's *t*‐test. * *p* < .05, ** *p* < .01 or *** *p* < .001 versus *ApoE^−/−^AnxA8^+/+^*. (D) P‐selectin, E‐selectin or CD31 concentrations in serum of *ApoE^−/−^AnxA8^+/+^* or *ApoE^−/−^AnxA8^−/^* ^−^ mice after fed HFD for 12 weeks (advanced lesions). Data represent mean ± SEM of 10 animals per group. Student's *t*‐test. ** *p* < .01 or *** *p* < .001 versus *ApoE^−/−^AnxA8^+/+^*.

Recruitment of leukocytes into arteries is a hallmark event in atherosclerosis progression. To assess whether changes in the expression of adhesion molecules can modulate leukocyte recruitment to activated ECs, we analysed the adhesion of PBMCs to *ApoE* ^−/−^ *AnxA8^+/+^* or *ApoE* ^−/−^ *AnxA8^−/^* ^−^ MAECs under static conditions. As expected, PBMCs adhesion was increased upon ox‐LDL stimulation in *AnxA8^+/+^* MAECs compared with unstimulated cells (Figure 8A). However, ox‐LDL failed to increase PBMCs adhesion in *AnxA8^−/−^* MAECs (Figure 8A). Lentiviral rescue of AnxA8 expression in *AnxA8^−/−^* MAECs recovered PBMCs‐endothelial adhesion after ox‐LDL stimulation (Figure S12).

To further analyse whether *AnxA8* deficiency impacts leukocyte recruitment in vivo, we compared the rolling and adhesion of leukocytes in the carotid arteries of *ApoE^−/−^AnxA8^+/+^* or *ApoE^−/−^AnxA8^−/−^* mice that had been fed an HFD for 4 weeks. In contrast to platelet adhesion that was consistently observed (Figure 6), the scarcity of spontaneous leukocyte−endothelial interactions with the carotid artery irrespective of the mouse genotype precluded any quantification. Due to this lack of quantifiable events in carotid arteries, we then compared the rolling and adhesion of leukocytes to calcium ionophore‐stimulated mesenteric venules between *ApoE^−/−^AnxA8^−/−^* or *ApoE^−/−^AnxA8^+/+^*. Intravital microscopy revealed that leukocyte rolling and adhesion were significantly diminished and leukocyte velocity was increased in *AnxA8*‐deficient mice as compared with control animals (Figure 8B and Movies S1 and S2).

Finally, E/P‐selectin and Pecam‐1 mRNA expression were diminished in the aorta of *ApoE^−/−^AnxA8^−/−^* compared with *ApoE^−/−^AnxA8^+/+^* mice (Figure 8C). In addition, circulating P/E selectin and CD31 concentrations were diminished in serum of *ApoE^−/−^AnxA8^−/−^* compared with *ApoE^−/−^ AnxA8^+/+^* mice in either the early (Figure S13) or the advanced model of atherosclerosis (Figure 8D). No significant differences were observed in VCAM‐1 or ICAM‐1 concentrations between serum of *ApoE^−/−^AnxA8^−/−^* compared with *ApoE^−/−^ AnxA8^+/+^* mice (Figure S10B). Overall, our results support the role of endothelial AnxA8 in leukocyte recruitment and adhesion to the injured vessel wall.

#### 3.6.1 Endothelial AnxA8 deficiency decreases atherosclerosis progression

**FIGURE 9:**
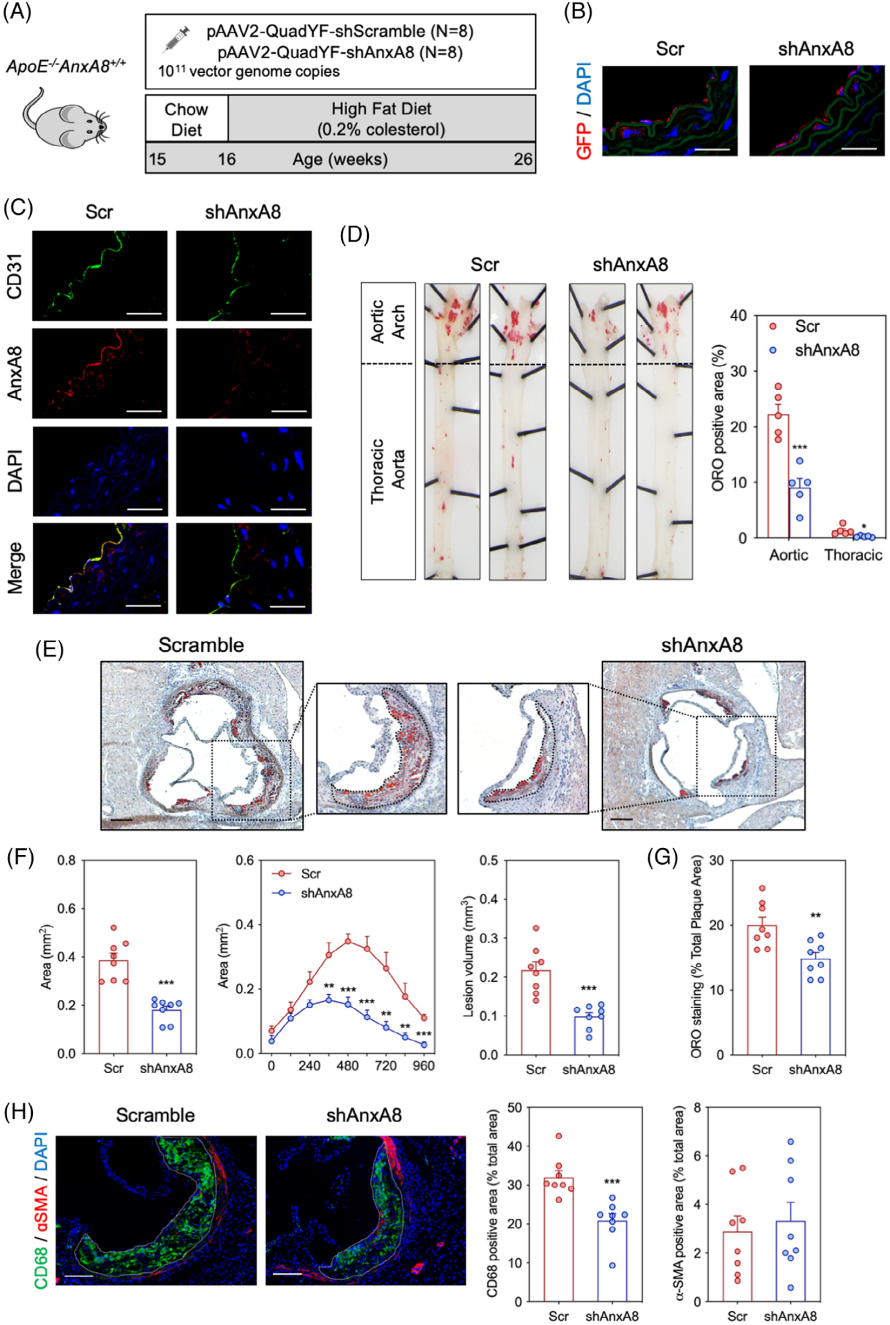
AnxA8 inhibition in endothelial cells reduces atherosclerosis progression. (A) Work flow for generation of endothelial *AnxA8*‐deficient animals in *ApoE^−/−^AnxA8^+/+^* mice fed with a high‐fat diet. (B) Representative immunofluorescence showing GFP expression in *ApoE^−/−^AnxA8^+/+^* mice injected with pAAV2‐QuadYF‐shScr or pAAV2‐QuadYF‐shAnxA8. Scale bars, 25 µm. (C) Representative immunofluorescence of AnxA8 (green) and their colocalization with ECs marker CD31 (red) or VSMCs marker α‐SMA (red) in aortic root sections of *ApoE^−/−^AnxA8^+/+^* mice injected with pAAV2‐QuadYF‐shScr or pAAV2‐QuadYF‐shAnxA8. Scale bars, 25 µm. (D) Representative pinned‐out *en face* aorta preparations and quantification of atherosclerosis from *ApoE^−/−^AnxA8^+/+^* mice injected with pAAV2‐QuadYF‐shScr or pAAV2‐QuadYF‐shAnxA8 stained with ORO after 10 weeks on HFD. Data represent mean ± SEM of five animals per group. * *p* < .05; *** *p* < .001 versus pAAV2‐QuadYF‐shScr. (E) Representative ORO/haematoxylin staining and quantification of maximal lesion area in the aortic root of *ApoE^−/−^AnxA8^+/+^* mice injected with pAAV2‐QuadYF‐shScr or pAAV2‐QuadYF‐shAnxA8 stained with ORO after 10 weeks on HFD. Data represent mean ± SEM of eight animals per group. Student's *t*‐test. ** *p* < .01; *** *p* < .001 versus pAAV2‐QuadYF‐shScr. Scale bars, 200 µm. (F) Quantification of atherosclerotic lesion area along aortic root in *ApoE^−/−^AnxA8^+/+^* mice injected with pAAV2‐QuadYF‐shScr or pAAV2‐QuadYF‐shAnxA8. The volume of atherosclerotic lesions was estimated by calculating the area under the curve for each condition. Data represent mean ± SEM of eight animals per group. Student's *t*‐test. ** *p* < .01; *** *p* < .001 versus pAAV2‐QuadYF‐shScr. (G) Quantification of ORO in the aortic root from *ApoE^−/−^AnxA8^+/+^* mice injected with pAAV2‐QuadYF‐shScr or pAAV2‐QuadYF‐shAnxA8 after 10 weeks on HFD. Data represent mean ± SEM of eight animals per group. ** *p* < .01 versus pAAV2‐QuadYF‐shScr. (H) Representative histological analysis of cross‐sections of the aortic sinus from *ApoE^−/−^AnxA8^+/+^* mice injected with pAAV2‐QuadYF‐shScr or pAAV2‐QuadYF‐shAnxA8 stained with CD68, α‐SMA and DAPI. Quantification of macrophages or VSMCs content are shown in the right panels. Scale bars, 100 µm. Data represent mean ± SEM of 10–11 animals per group. Student's *t*‐test. *** *p* < .001 versus pAAV2‐QuadYF‐shScr.

To analyse the contribution of endothelial AnxA8 in the progression of atherosclerosis, we used pAAV2‐QuadYF, an adenovirus that shows a specific tissue tropism for ECs and retina.^34^ Fifteen week‐old atheroprone *ApoE^−/−^* mice were given retro‐orbital injections of pAAV2‐QuadYF‐shScramble or pAAV2‐QuadYF‐shAnxA8. One week after the AAV injection, animals were fed on HFD for 10 weeks (Figure 9A). Green Fluorescence Protein (GFP) expression was specifically expressed in ECs (Figure 9B). In addition, pAAV2‐QuadYF‐shAnxA8 efficiently inhibited AnxA8 expression in ECs (Figure 9C). No differences were observed in body weight or serum cholesterol, triglycerides, HDL‐c or LDL‐c concentrations between the different groups (Figure S14A,B). We assessed the atherosclerotic lesions in the entire aorta by *en face* staining with ORO to visualize lipid‐laden atherosclerotic plaques. We found a 60% reduction of the *en face* aortic arch lesion area in *ApoE^−/−^* mice transduced with shAnxA8 compared to shScr‐transduced mice (Figure 9D). In addition, a marked reduction was observed in the atherosclerotic lesion size and volume at the aortic root of shAnxA8 transduced *ApoE^−/−^* mice compared to shScr mice (≈ 55% reduction for both; Figure 9E,F). We also observed a marked reduction in lipid deposition (Figure 9E,G) without changes in collagen content in the aortic wall of shAnxA8‐*ApoE^−/−^* mice treated with compared to shScr‐*ApoE^−/−^* mice (Figure S14C). Analysis of atheroma also revealed decreased accumulation of CD68^+^ cells, without affecting smooth muscle cell content in the shAnxA8 transduced *ApoE^−/−^AnxA8^−/−^* mice compared to the shScr group (Figure 9H). Collectively, these results demonstrate that endothelial AnxA8 inhibition attenuates plaque progression in atheroprone mice.

## 4 DISCUSSION

Atherosclerosis is a common pathological aetiology of CVD.^35^ To improve our understanding of the molecules involved in atherogenesis, we used RNA‐Seq to systematically investigate the global transcriptome of atherosclerotic aortas from *ApoE^−/^* ^−^ mice compared to WT mice. As expected, we have observed an upregulation of genes related to T cell differentiation and the adaptive immune system in the aorta of *ApoE^−/−^* mice compared to WT mice. Adaptive immunity plays a critical role in the development and progression of atherosclerosis. T cell functions affect the balance between progression and resolution of plaque inflammation. Depending on the cytokine microenvironment, naïve CD4^+^ T cells can differentiate into specialized effector cells (Th1, Th2, Th17, etc.), being Th1 cells a major source of proatherogenic cytokines such as interferon‐gamma and tumour necrosis factor.^36^ However, T regulatory cells produce anti‐inflammatory IL‐10, transforming growth factor β and IL‐35, preventing inflammation and plaque progression.^37^

We also identified *AnxA8* as one of the most significantly upregulated genes in atherosclerotic aortas of *ApoE^−/−^* mice. In agreement, Wierer et al. observed by proteomic analysis that AnxA8 was upregulated in the aorta of *ApoE^−/−^* mice compared to the aorta of WT animals.^38^ The finding that the germline AnxA8 deficiency drastically reduces the atherosclerotic burden in both early and advanced atherosclerosis in an *ApoE^−/−^* mouse model argues for a functional role of AnxA8 in the progression of atherosclerosis. Although we are unknown whether other annexins expression could be modulated in AnxA8‐deficient mice, their modulation did not counter the beneficial effect observed in AnxA8‐deficient mice. The loss of effect of *AnxA8*‐deficient BMT in *ApoE*‐deficient mice suggests a direct role for AnxA8 in resident vascular cells, consistent with microarray studies suggesting that AnxA8 is poorly expressed in murine leukocytes.^39^

The delayed progression of atherosclerosis observed in *AnxA8*‐deficient mice appears to be the result of reduced inflammatory cell content in plaques. In response to hyperlipidaemia, the BM and spleen overproduce pro‐inflammatory monocytes that can infiltrate, accumulate and differentiate into macrophages in atherosclerotic lesions.^40^ We observed a reduction in CD68^+^ cells in AnxA8‐deficient mice, which was associated with a decrease in atherosclerotic plaque size and volume, and serum cytokine levels. In addition, AnxA8‐deficient animals develop more stable atherosclerotic plaques with a smaller necrotic core, thicker fibrous cap, less lipid accumulation and apoptosis, and increased contractile and decreased synthetic VSMC markers expression. Accordingly, plaque complexity of AnxA8‐deficient mice decreased in both early and advanced models. However, our data show that AnxA8 does not play a direct role in VSMC phenotypic switching or macrophage lipid metabolism, supporting the potential role of endothelial AnxA8 in atherosclerosis. Oxidized LDL activates ECs by promoting the expression and secretion of a variety of adhesion molecules and chemotactic factors at atherosclerosis‐prone sites,^41^ and we have shown that ox‐LDL upregulates AnxA8 expression in ECs in a time‐ and dose‐dependent manner. The upregulation of AnxA8 by ox‐LDL may be related to the long non‐coding RNA taurine‐upregulated gene 1 (TUG1), a lncRNA that is actively involved in various physiological processes, including the regulation of genes at epigenetic, transcription, post‐transcription, translation and post‐translation levels. TUG‐1 is known to promote AnxA8 expression and ox‐LDL increases TUG‐1 expression in ECs.^42^, ^43^ However, this hypothesis needs to be tested.

AnxA8 controls the pathway that ensures the sorting and delivery of late endosomal CD63 to WPB. Once the endothelium is activated by inflammatory mediators, CD63 is transported to WBP, which contain P‐selectin among other proteins. Then, WBP fuse with the plasma membrane, allowing P‐selectin to be presented on the cell surface, along with its stabilizing co‐factor CD63.^15^ Increased WPB secretion and P‐selectin in the ECs membrane promotes platelet‐ECs adhesion, and hypercholesterolemia increases P‐selectin‐dependent platelet‐ECs adhesion in mice.^44^ Activated platelets exacerbate atherosclerosis by recruiting monocytes and other leukocytes.^45^ In addition, platelets may also interact with the activated endothelium prior to the formation of the atherosclerotic plaque, which plays a role in the pathogenesis of atherosclerosis.^46^ In this context, our in vitro experiments under continuous flow showed reduced platelet adhesion on ox‐LDL‐stimulated *AnxA8*‐deficient ECs. These results were confirmed by in vivo intravital microscopy on carotid arteries with incipient lesions in *AnxA8*‐deficient mice, which clearly showed reduced platelet aggregation. Platelets also express and store P‐selectin in α‐granules,^47^ and it has been reported that P‐selectin‐deficient platelets fail to adhere to ECs in hypercholesterolemic mice.^44^ The diminution of platelet adhesion to AnxA8‐deficient ECs could be due to a deficiency in P‐selectin expression or surface presentation in AnxA8‐deficient platelets. However, our in vitro experiments indicated that under thrombolytic stimulus, *AnxA8*‐deficient platelets show no differences in P‐selectin expression, and platelets from *ApoE^−/−^AnxA8^−/−^* adhered to *AnxA8^+/+^* MAECs pre‐stimulated with ox‐LDL, suggesting that platelet P‐selectin content and trafficking are not affected by AnxA8 deficiency in platelets.

P‐ and E‐selectins are expressed in chronically inflamed endothelium and serve as rolling molecules for monocytes, neutrophils, effector T cells, B cells and natural killer cells. Leukocyte rolling is mainly achieved by the interaction of endothelial E‐ and P‐selectin with PSGL‐1 and other glycosylated ligands such as CD44 and E‐selectin ligand 1 (ESL‐1), which specifically bind to E‐selectin on leukocytes.^48^, ^49^ While E‐selectin deficiency alone has little effect on the progression of atherosclerotic plaque formation, P‐selectin deficiency has a greater impact on atherosclerotic development.^50^ Furthermore, combined gene silencing of P‐ and E‐selectin in *LDLR*‐deficient mice also reduces diet‐induced atherosclerotic lesion progression in both the aortic sinus and the whole aorta.^51^ Under atherogenic stimuli such as ox‐LDL (in vitro) or high‐fat diet (in vivo), we observed that AnxA8 deficiency prevented P‐ and E‐selectin expression and secretion in ECs and mouse serum. Furthermore, leukocyte adhesion to ox‐LDL‐activated endothelium was reduced in AnxA8‐deficient ECs, which was validated in mesenteric veins of atheroprone *AnxA8^−/^* ^−^ mice, with a slight leukocyte adhesion and increased leukocyte rolling velocities.

Platelet endothelial cell adhesion molecule‐1, also known as CD31, is expressed at high densities on the lateral borders of ECs and is upregulated at the atherosclerotic‐prone sites and also regulates leukocyte accumulation within the arterial wall.^52^ After leukocyte rolling, leukocytes need to adhere firmly to the endothelium to continue transendothelial migration in which leukocytes move across the endothelium, involving dynamic signalling between leukocytes and ECs.^53^ Ox‐LDL directly promotes monocyte transmigration across cytokine‐stimulated ECs by a mechanism involving upregulation of EC CD31 in the basolateral membranes.^54^ In addition, genetic deletion of CD31 resulted in significantly reduced atherosclerotic lesion size in *ApoE*‐deficient mice.^55^ Consistently, ox‐LDL upregulated CD31 expression and secretion in ECs, which was prevented in *AnxA8*‐deficient ECs. In vivo results also suggest a role for AnxA8 in modulating transendothelial cell migration, as *AnxA8^−/−^* mice showed less inflammatory content in atheroma lesions, and CD31 serum concentrations were also reduced in *AnxA8^−/−^* compared with *AnxA8^+/+^* mice. Although other adhesion molecules play an important role in leukocyte recruitment to the injured arteries,^52^ no differences in ICAM‐1 or VCAM‐1 expression and secretion between *AnxA8^−/−^* and *AnxA8^+/+^* ECs were observed.

The precise molecular mechanism involved in the regulation of adhesion molecule expression by endothelial AnxA8 has not yet been identified. P‐selectin is prestored in WBP and translocated to the luminal membrane in response to inflammatory stimuli, whereas E‐selectin is de novo synthesized upon cell activation.^56^ It has been shown that CD31 is concentrated at surface vesicular membrane invaginations and that during transendothelial cell migration, it is moved to the border areas where migration occurs. However, recycling of CD31 occurs and the precise mechanisms that trigger this response are unclear.^57^ In ECs, the Akt signalling pathway has been shown to be involved in the expression of endothelial adhesion molecules.^32^, ^33^ We have now observed that ox‐LDL induces Akt phosphorylation in ECs, an effect that is at least partially prevented in AnxA8‐deficient cells. Consistently, AnxA8 also activates Akt phosphorylation in endometrial cells.^58^

Finally, endothelial‐specific inhibition of AnxA8 mimics the effects observed in germline deficient mice including the diminution of atherosclerotic lesion size and volume, as well as lower lipid and inflammatory cell content within atherosclerotic plaques. Altogether, our results indicate an essential role of endothelial AnxA8 in atherosclerosis progression.

The study of KO animals is limited in terms of possible mechanisms of compensation from birth that can impact the effects of germline deletion. In our study, we cannot discard that in our AnxA8 null mice, there are other upregulated proteins and/or compensatory mechanisms that could influence the results observed. However, it is important to note that specific inhibition of AnxA8 in ECs mimics all effects observed in germline‐deficient mice. Further studies are needed to fully elucidate the mechanisms of AnxA8 upregulation and contributions to inflammatory diseases.

## 5 CONCLUSION

We have shown an upregulation of AnxA8 in both human and murine atherosclerotic plaques, identifying AnxA8 as an important mediator of atherosclerosis. Our results strongly support that AnxA8 promotes disease progression by regulating adhesion molecules expression and the influx of immune cells to the intima. The findings of our study suggest that interventions capable of reducing AnxA8 expression in ECs may delay atherosclerotic plaque progression.

## AUTHOR CONTRIBUTIONS

J.L.M.‐V., U.R., N.M.‐B. and L.M.B.‐C. contributed to the study design. G.N.‐M. and P.M. performed RNA‐Seq and analysed the obtained data. V.O. and B.H.‐T.N. performed intravital microscopy. C.G.‐M., R.B.‐S., I.S.S.‐J., S.S.‐A., M.J.F.‐G., P.N., J.C.E.‐G., N.M.‐B. and L.M.B.‐C. performed the experiments. B.H.‐T.N., J.L.M.‐V., N.M.‐B. and L.M.B.‐C. analysed and interpreted the data. J.L.M.‐V., U.R., J.C.E.‐G., B.H.‐T.N., N.M.‐B. and L.M.B.‐C. wrote and revised the manuscript. All authors reviewed and proofread the manuscript. All authors read and approved the final manuscript.

## CONFLICT OF INTEREST STATEMENT

The authors declare that the research was conducted in the absence of any commercial or financial relationships that could be construed as a potential conflict of interest.

## ETHICS STATEMENT

This study was approved by the Ethical Committee of the French Biomedicine Agency and written consent was obtained from all patients. All animal procedures were performed in accordance with the guidelines of Directive 2010/63/EU of the European Parliament on the protection of animals used for scientific purposes and were approved by the Institutional Animal Care and Use Committee and Comunidad de Madrid (PROEX 238.0/20).

## Supporting information



Supporting Information

Supporting Information

Supporting Information

Supporting Information

Supporting Information

Supporting Information

Supporting Information

Supporting Information

Supporting Information

Supporting Information

Supporting Information

Supporting Information

Supporting Information

Supporting Information

Supporting Information

Supporting Information

Supporting Information

## Data Availability

The data that support the findings of this study are available from the corresponding author upon reasonable request. RNA‐seq datasets have been deposited in NCBIGene Expression Omnibus and are accesible through GEO series number GSE207414.
