# Supplementary figures and images for: Annexin A8 deficiency delays atherosclerosis progression
Source: Clin Transl Med. 2025 Jan 21;15(1):e70176. doi: 10.1002/ctm2.70176 (PMC11748212; doi:10.1002/ctm2.70176)

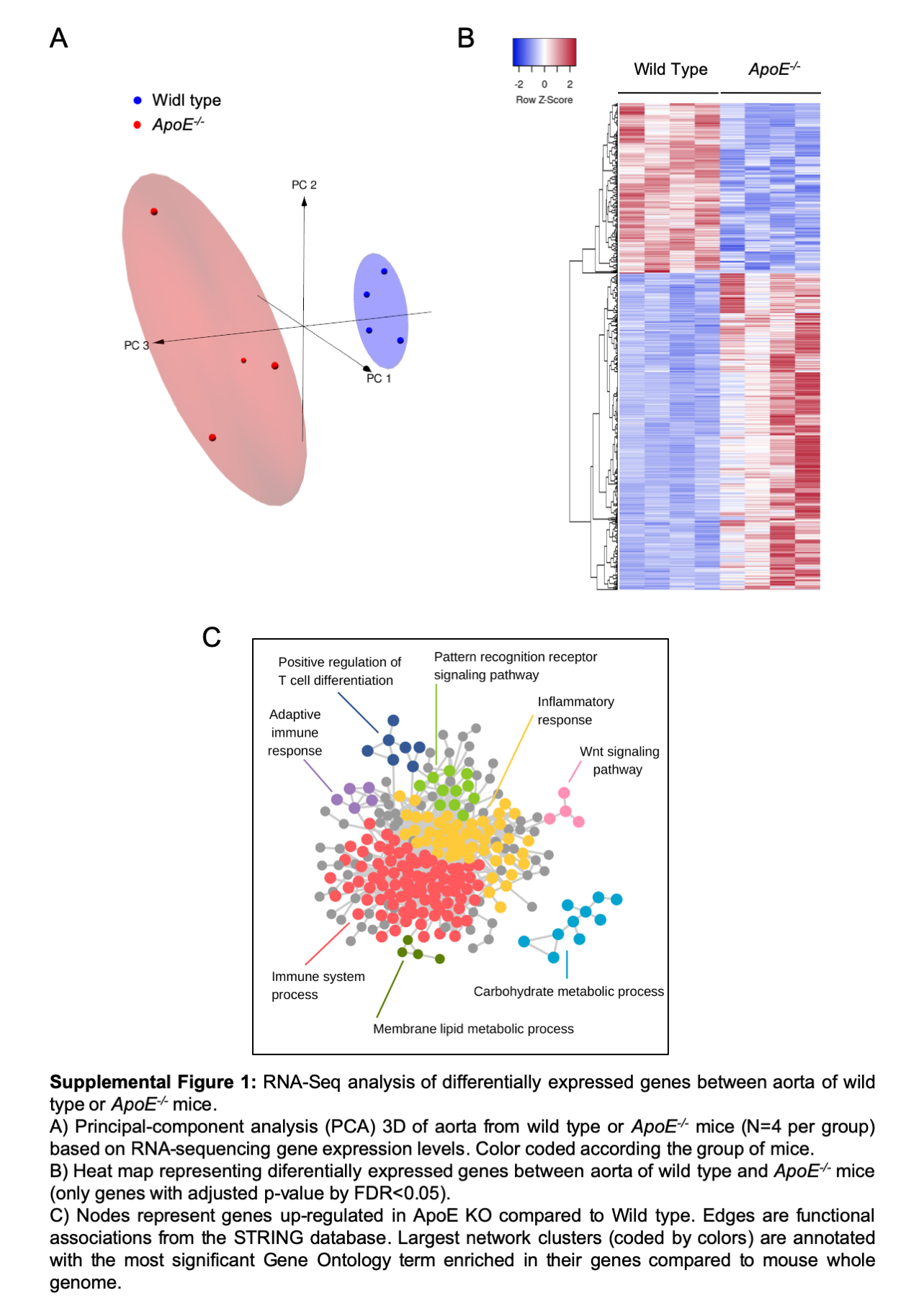

Supplement: Supplementary file 1 — Supporting Information [file CTM2-15-e70176-s016.tiff]

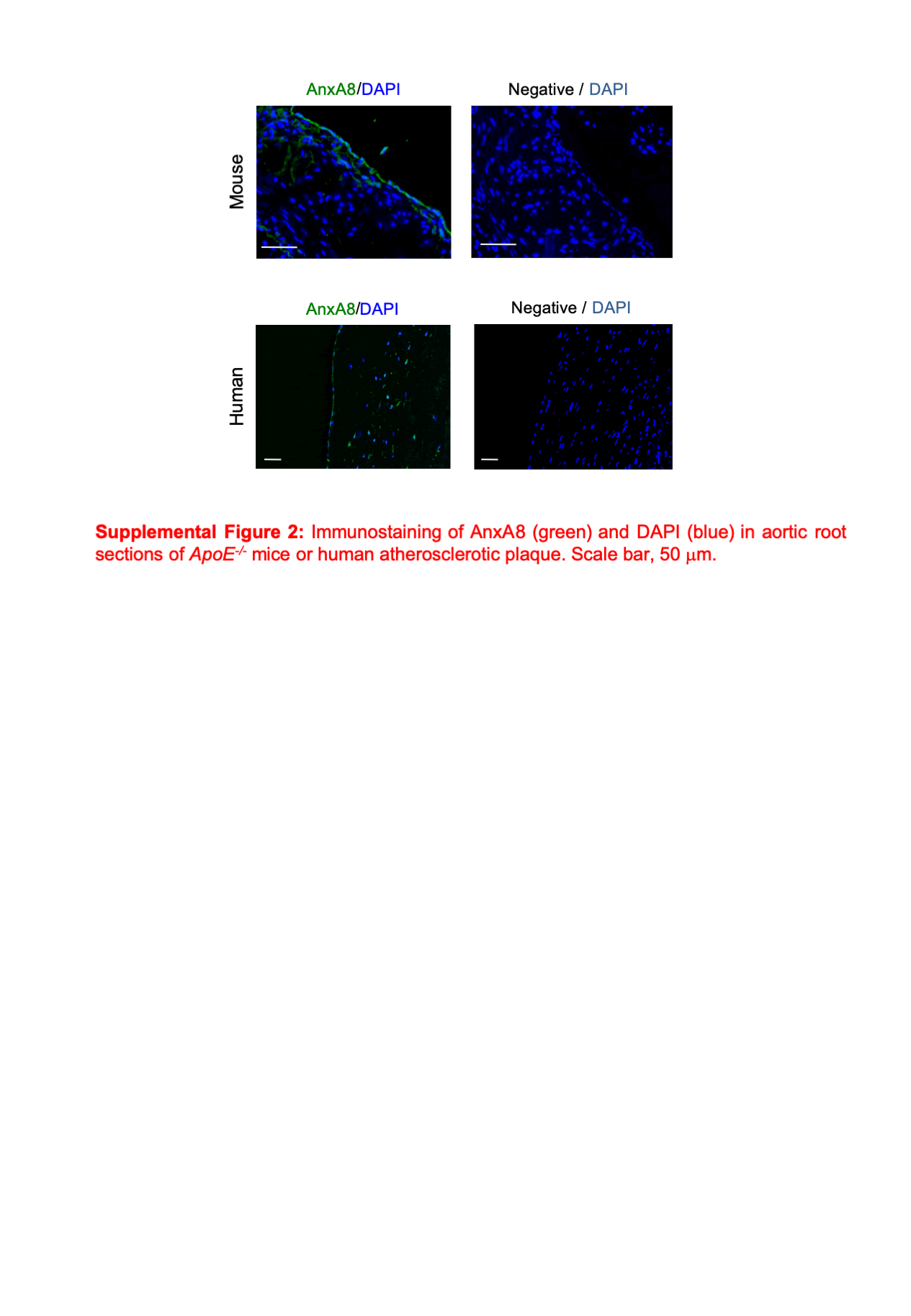

Supplement: Supplementary file 2 — Supporting Information [file CTM2-15-e70176-s006.tiff]

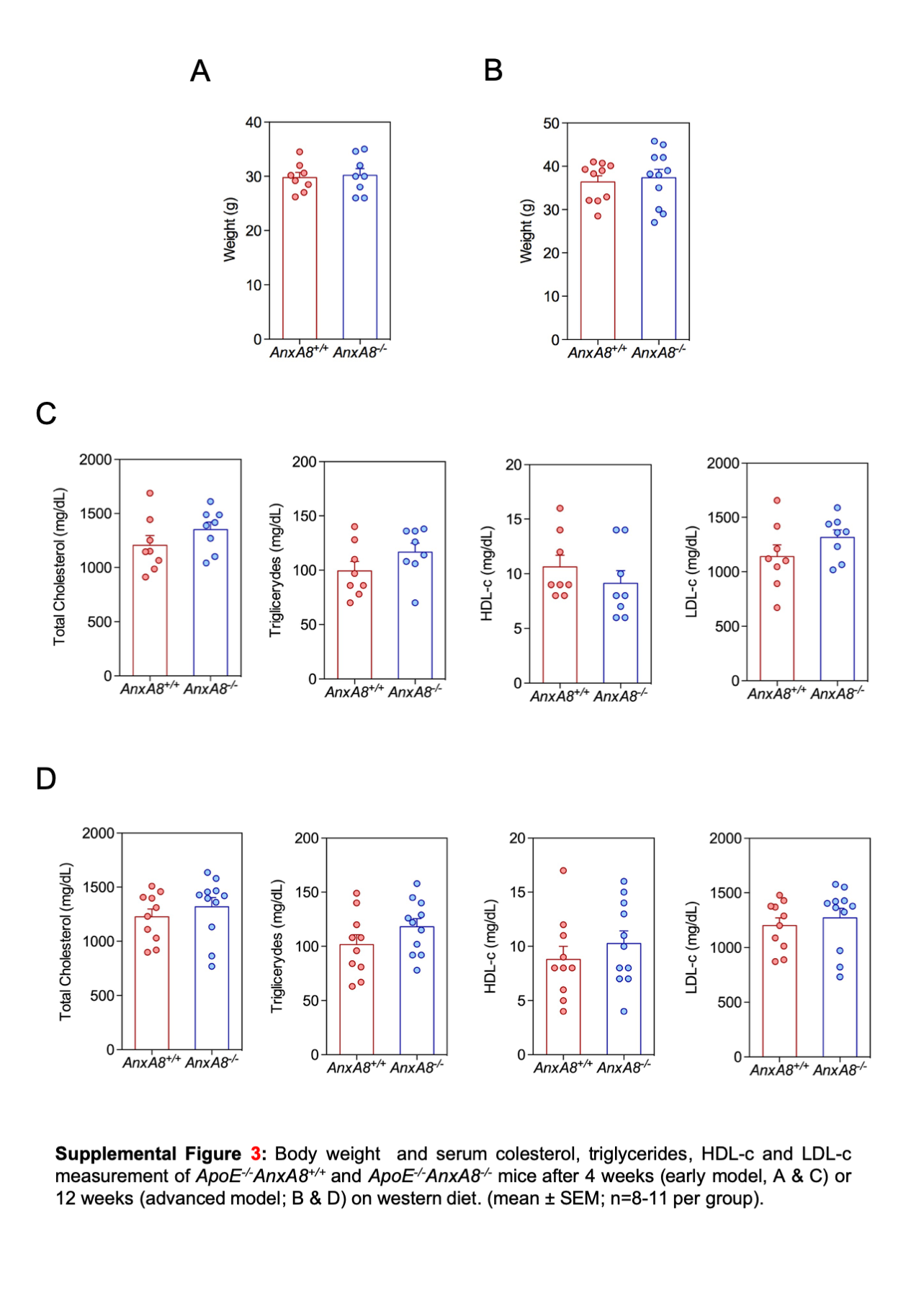

Supplement: Supplementary file 3 — Supporting Information [file CTM2-15-e70176-s012.tiff]

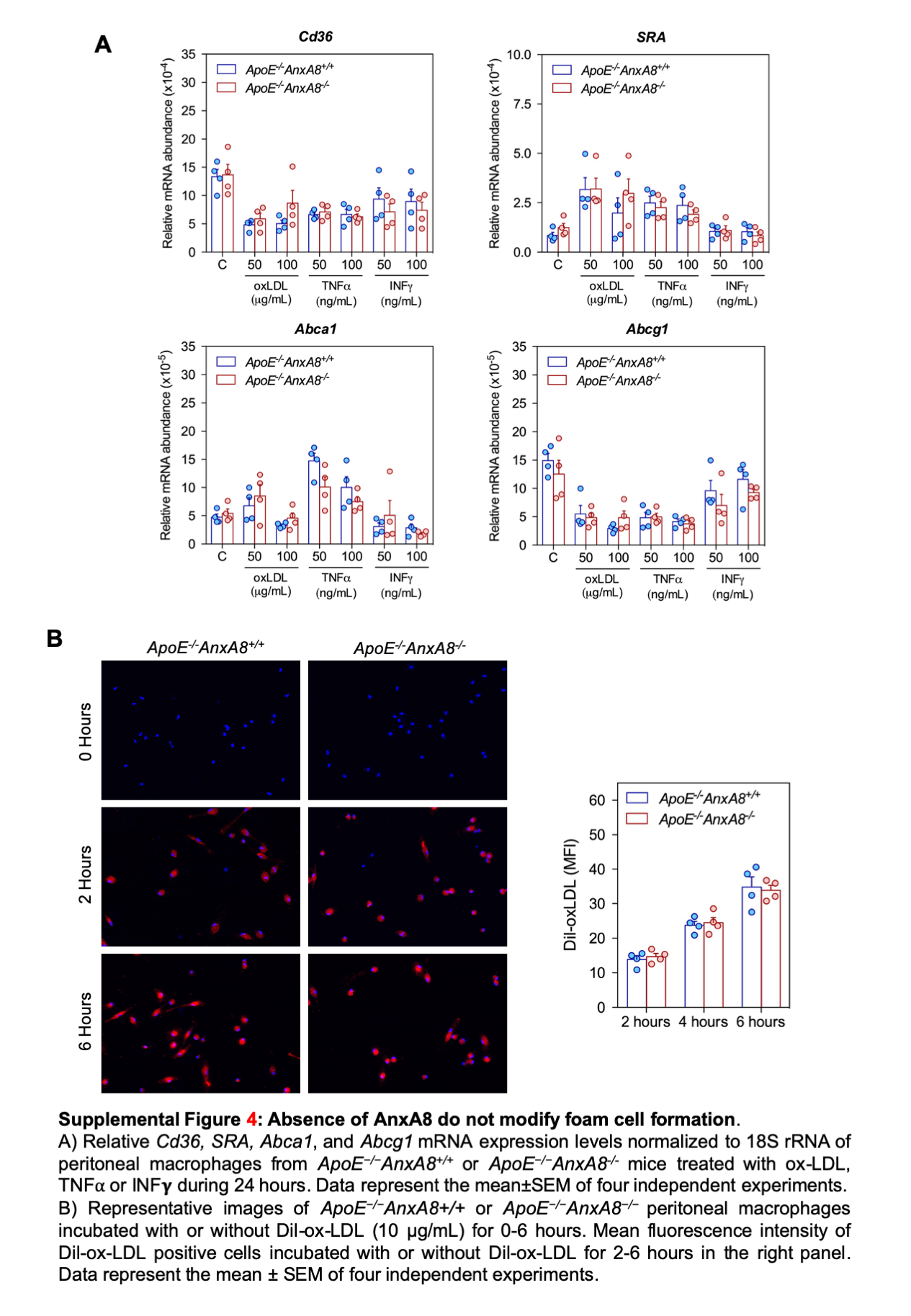

Supplement: Supplementary file 4 — Supporting Information [file CTM2-15-e70176-s008.tiff]

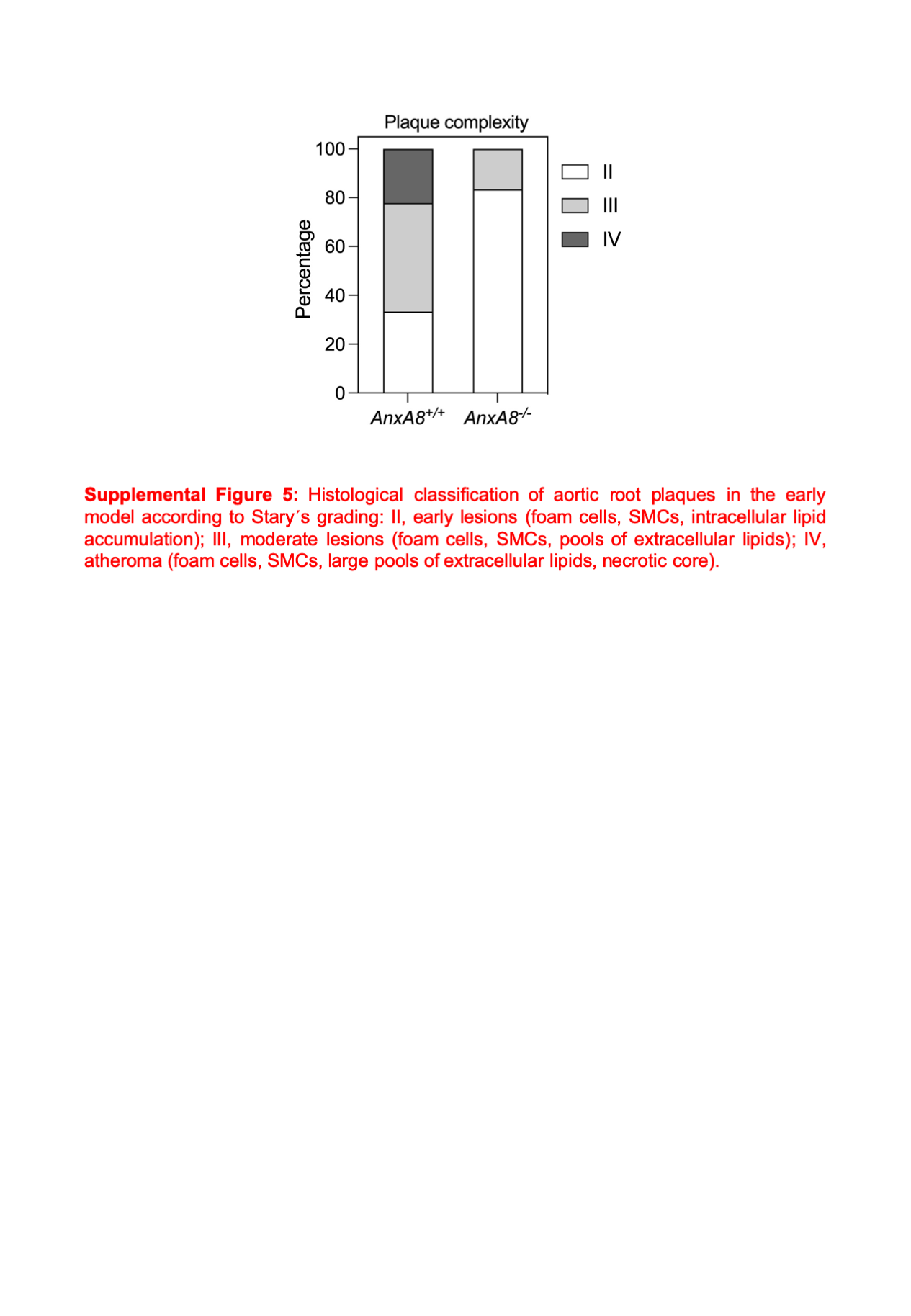

Supplement: Supplementary file 5 — Supporting Information [file CTM2-15-e70176-s001.tiff]

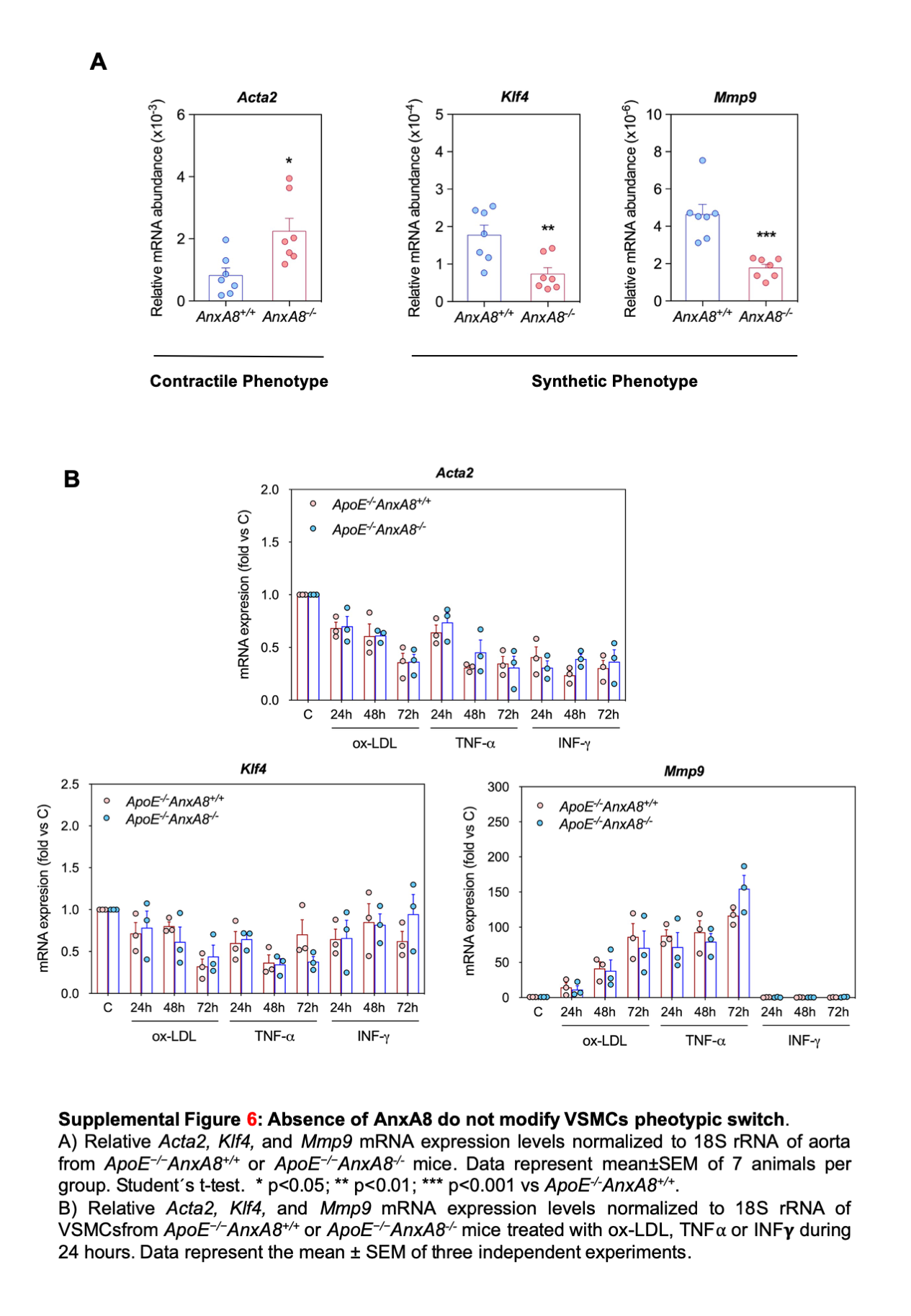

Supplement: Supplementary file 6 — Supporting Information [file CTM2-15-e70176-s005.tiff]

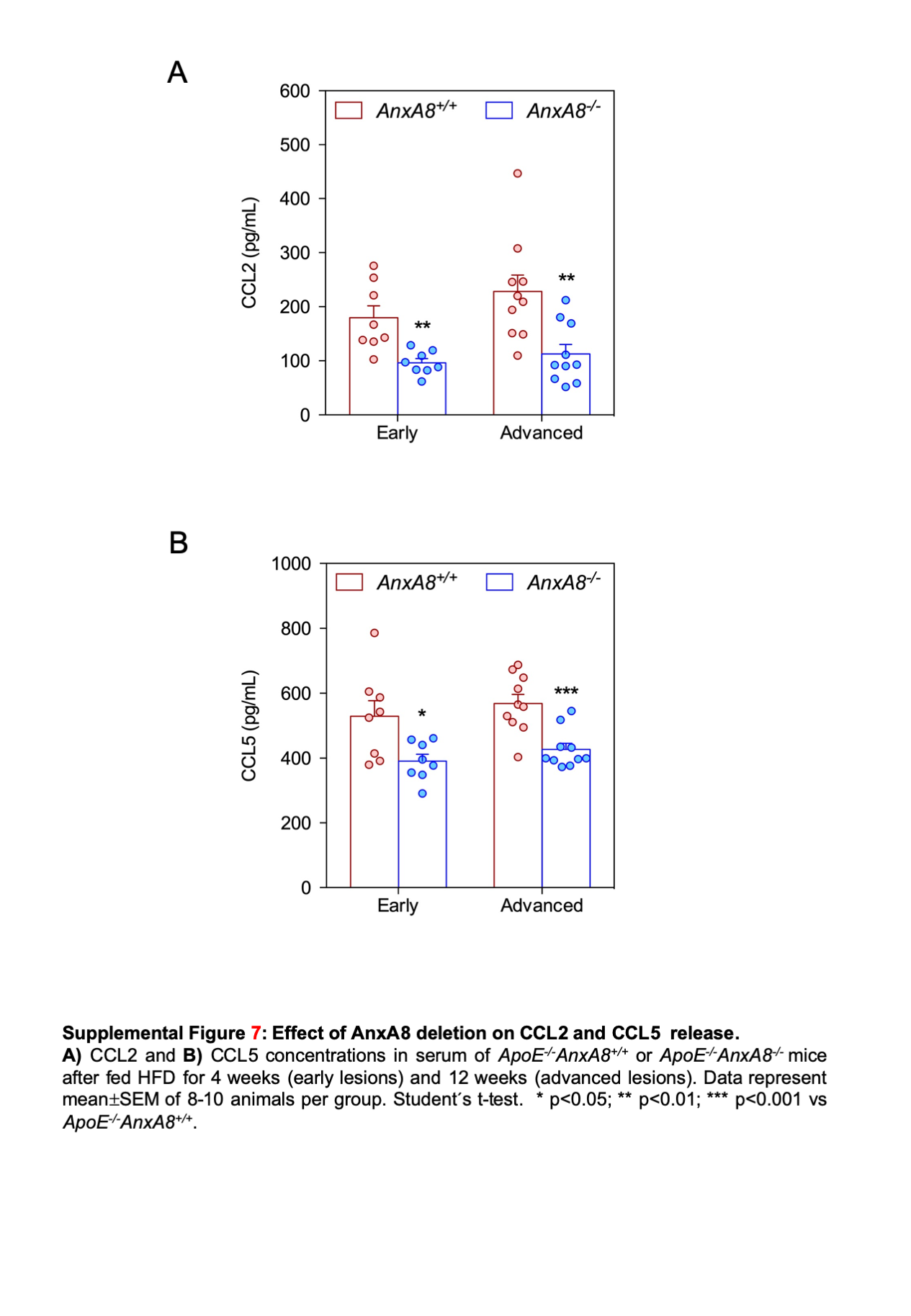

Supplement: Supplementary file 7 — Supporting Information [file CTM2-15-e70176-s002.tiff]

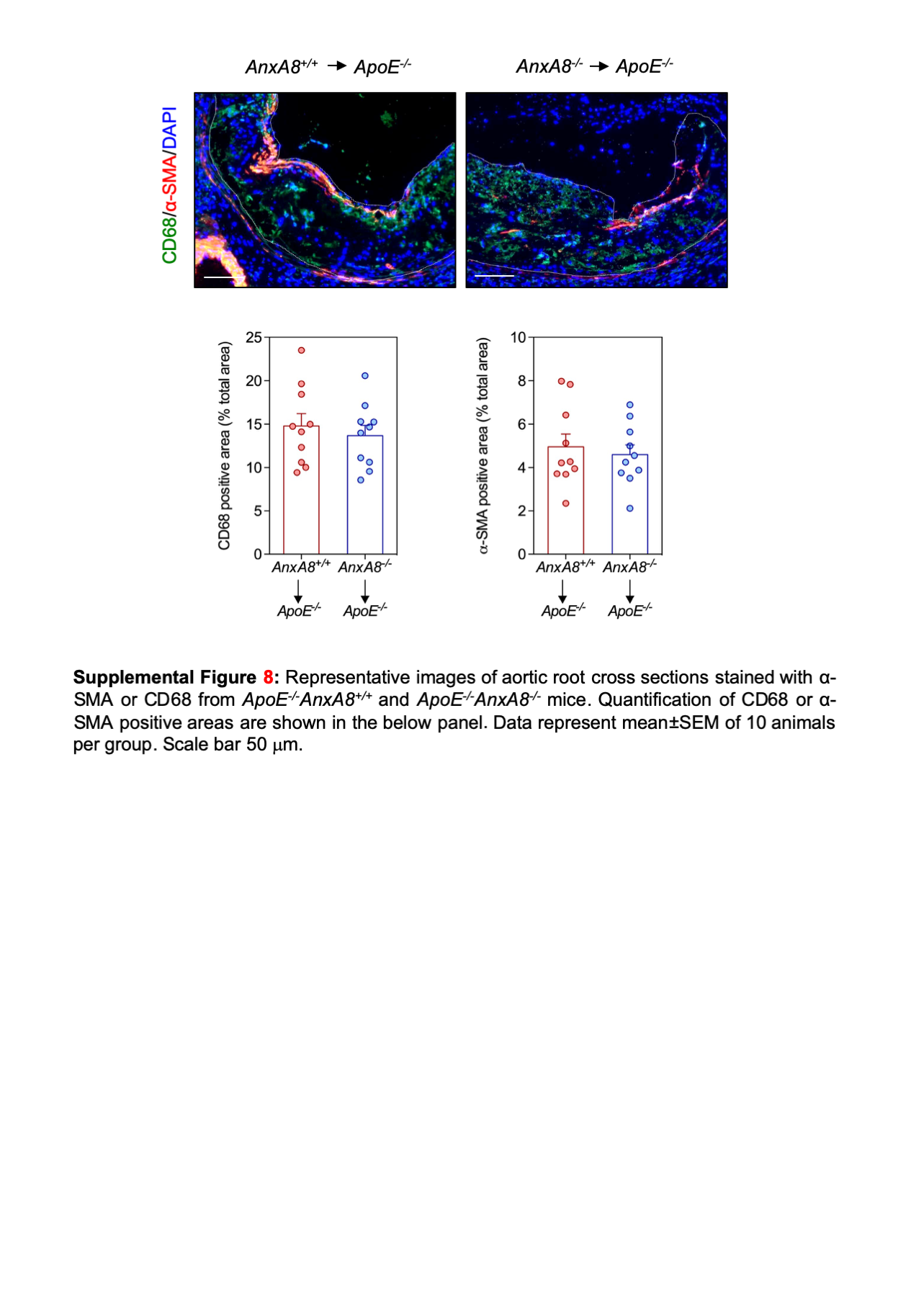

Supplement: Supplementary file 8 — Supporting Information [file CTM2-15-e70176-s010.tiff]

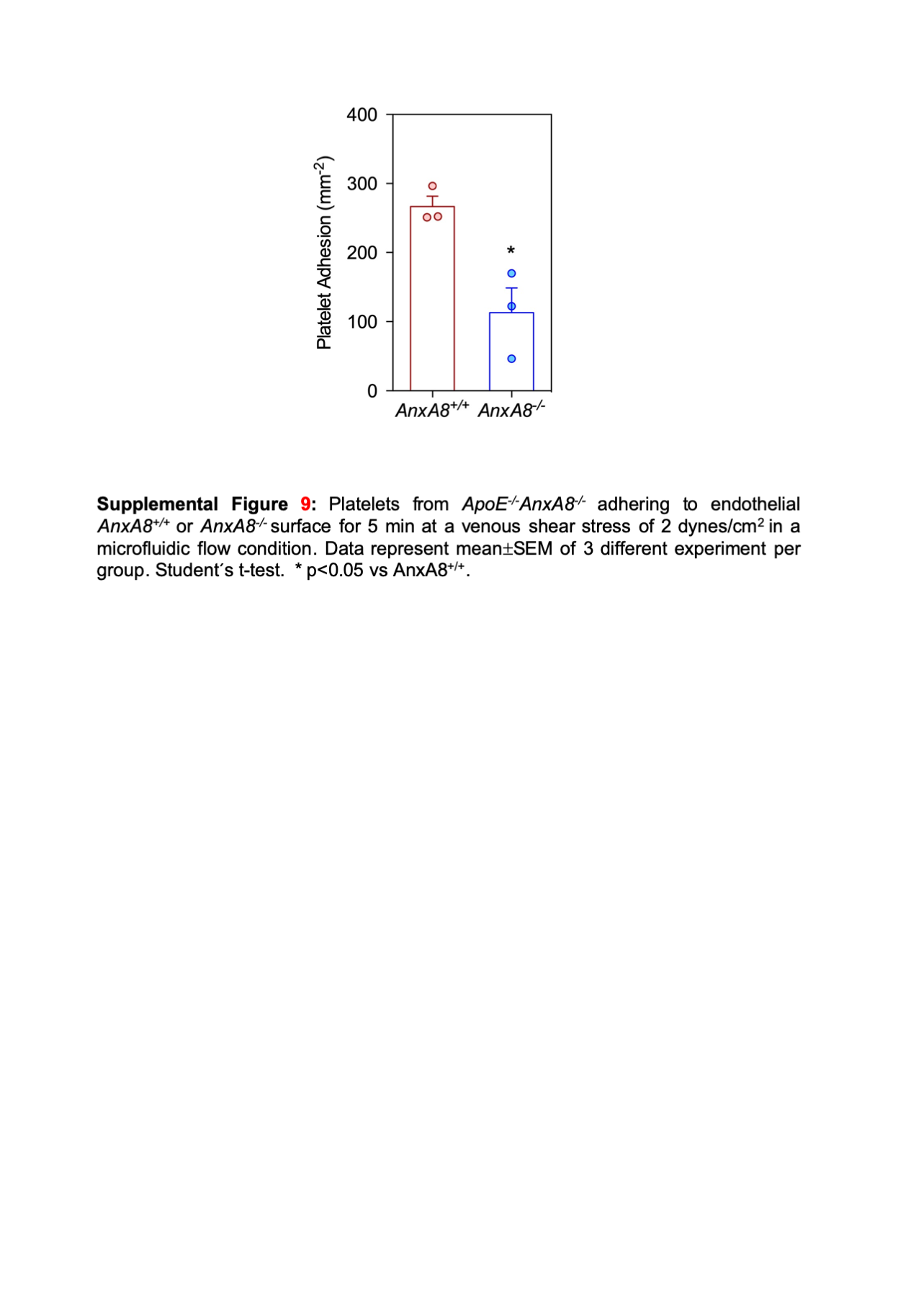

Supplement: Supplementary file 9 — Supporting Information [file CTM2-15-e70176-s015.tiff]

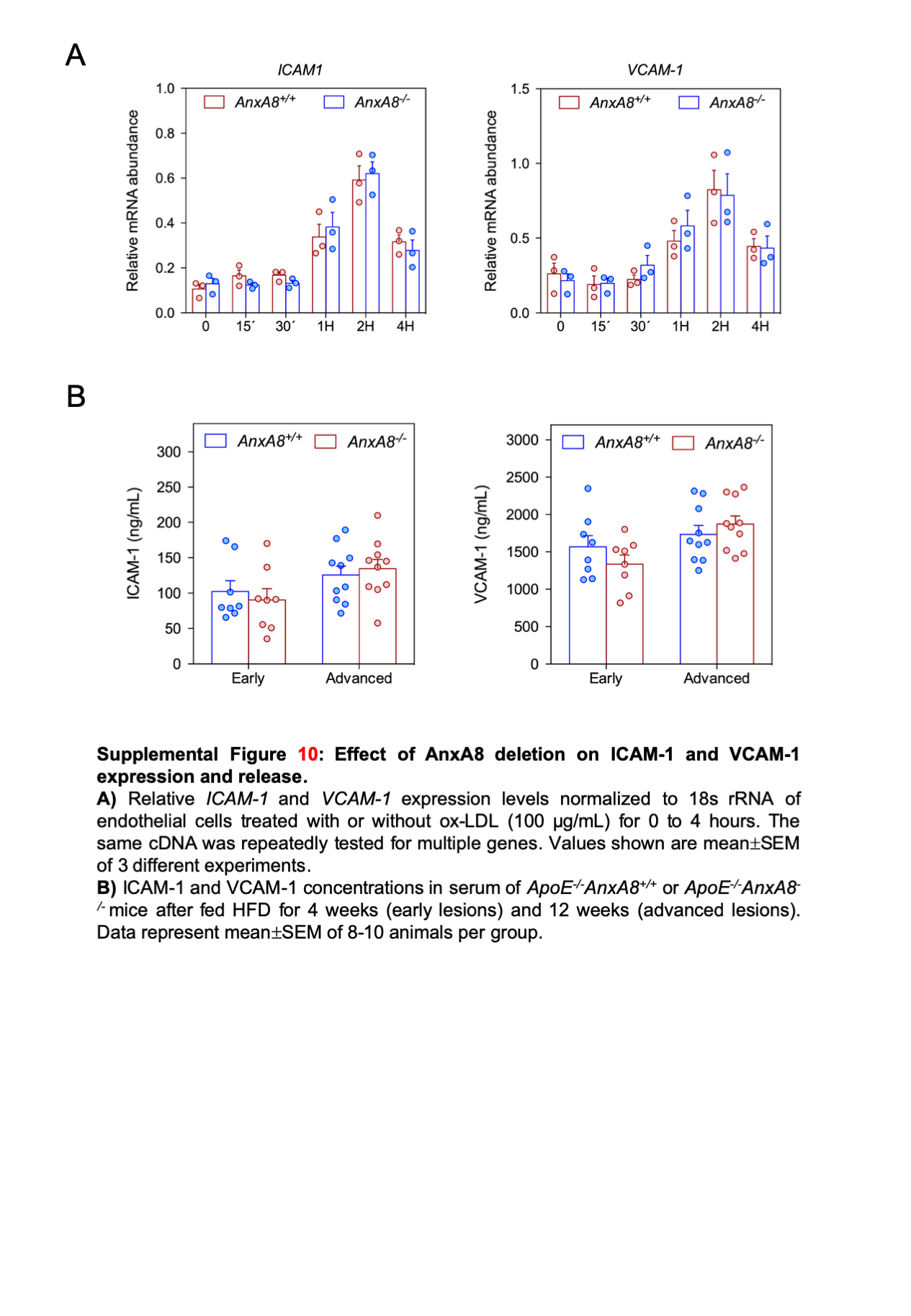

Supplement: Supplementary file 10 — Supporting Information [file CTM2-15-e70176-s017.tiff]

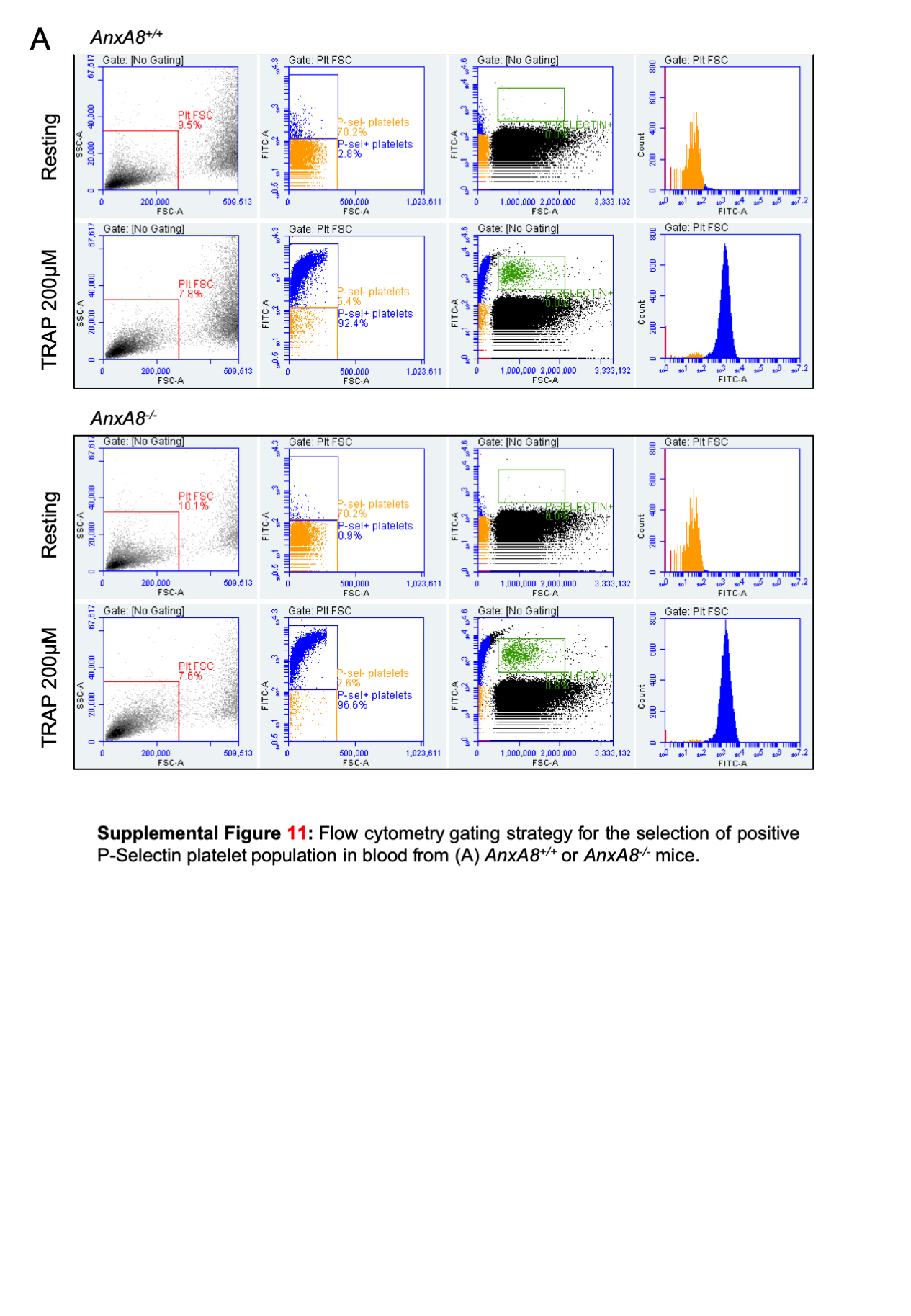

Supplement: Supplementary file 11 — Supporting Information [file CTM2-15-e70176-s014.tiff]

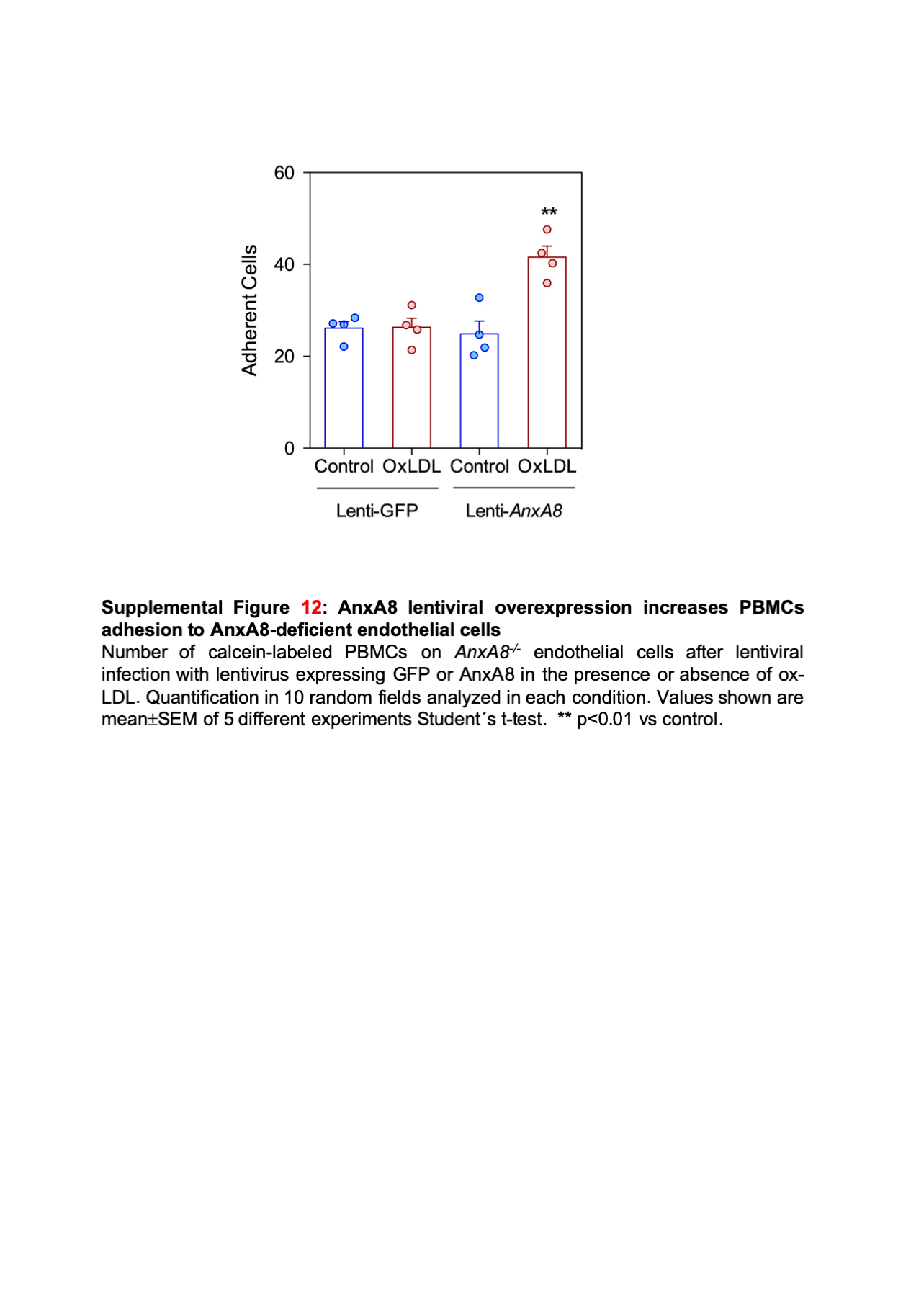

Supplement: Supplementary file 12 — Supporting Information [file CTM2-15-e70176-s013.tiff]

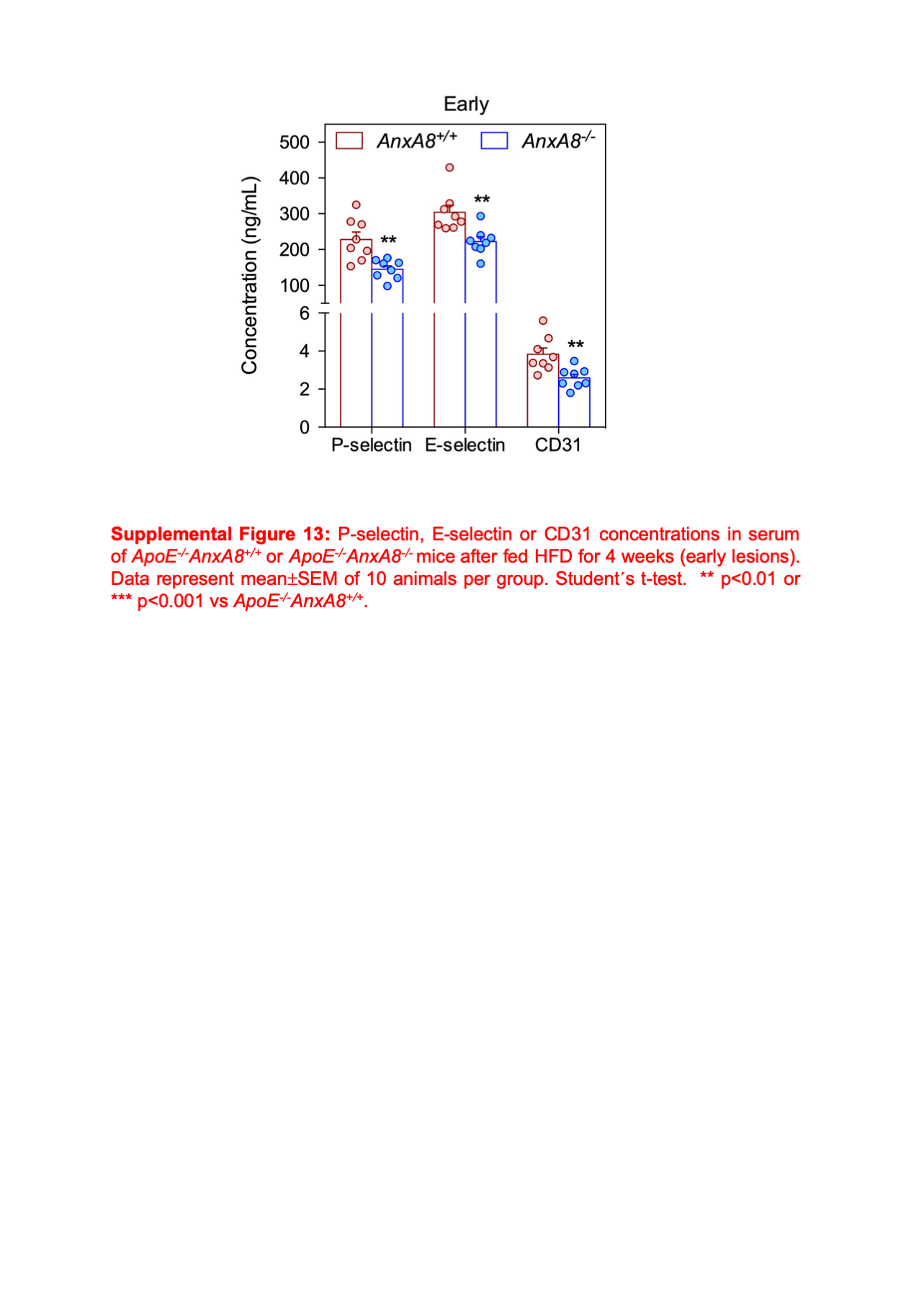

Supplement: Supplementary file 13 — Supporting Information [file CTM2-15-e70176-s007.tiff]

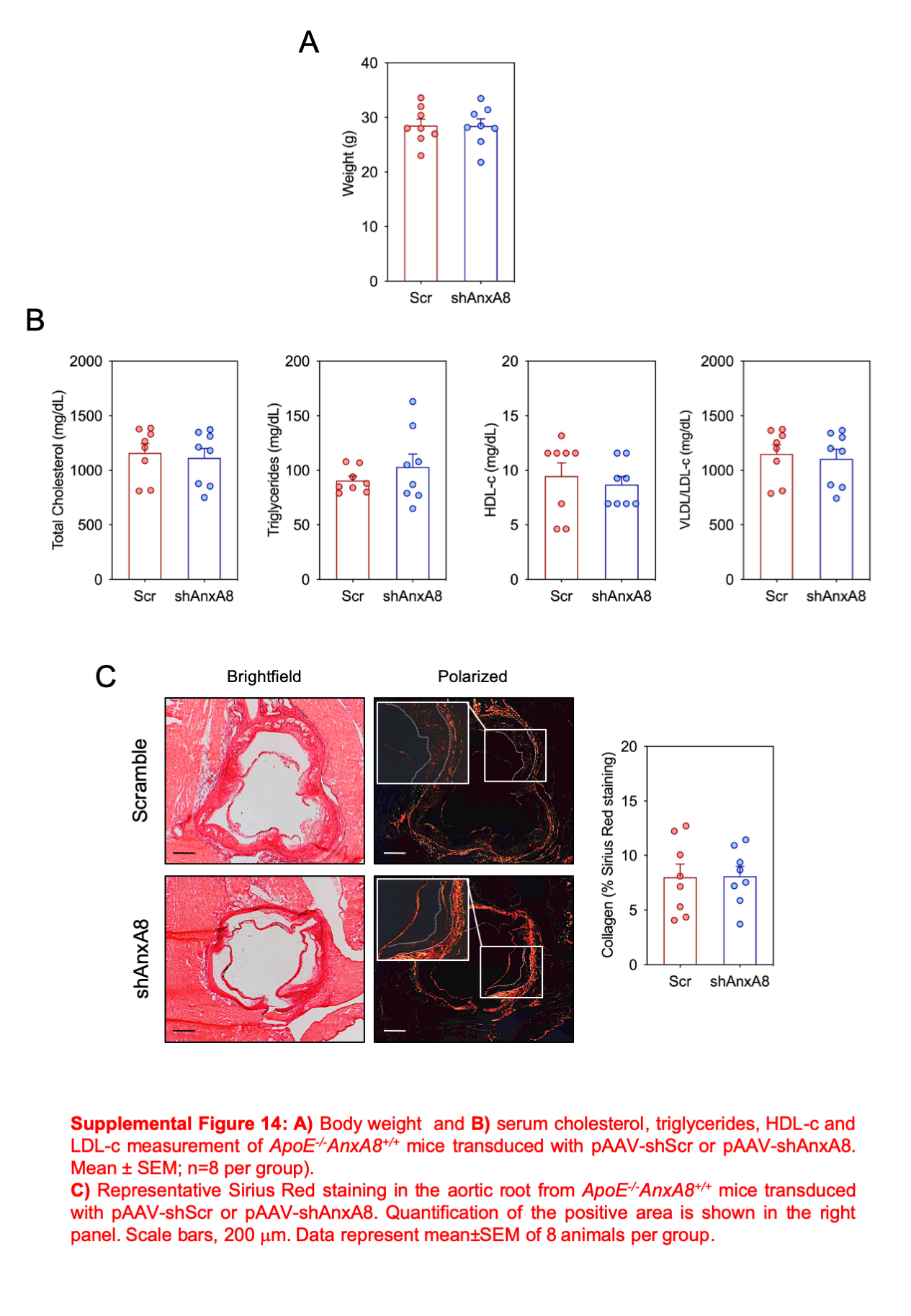

Supplement: Supplementary file 14 — Supporting Information [file CTM2-15-e70176-s009.tiff]
