# Supplementary material for: Annexin A8 deficiency delays atherosclerosis progression
Source: Clin Transl Med. 2025 Jan 21;15(1):e70176. doi: 10.1002/ctm2.70176 (PMC11748212; doi:10.1002/ctm2.70176)
Supplement: Supplementary file 15 — Supporting Information [file CTM2-15-e70176-s004.docx]

**Supplemental Table 1.** Gene expression assays

| **qPCR primers** | **Forward** | **Reverse** |
| --- | --- | --- |
| *Anxa8* | 5´-CGTCAGCGTGAAGGGTAGTTC-3´ | 5´-CTTGGTGAGCAGTCTATGATG-3´ |
| *Cdkn1c* | 5´-CGAGGAGCAGGACGAGAATC-3´ | 5´-GAAGAAGTCGTTCGCATTGGC-3´ |
| *Pecam1* | 5´-ACGCTGGTGCTCTATGCAAG-3´ | 5´-TCAGTTGCTGCCCATTCATCA-3´ |
| *Selp* | 5´-GAAAGGGCTGATTGTGACCCC-3´ | 5´-AGTAGTTCCGCACTGGGTACA-3´ |
| *Sele* | 5´-ATGCCTCGCGCTTTCTCTC-3´ | 5´-GTAGTCCCGCTGACAGTATGC-3´ |
| *Acta2* | 5´-CATCTTTCATTGGGATGGAG-3´ | 5´-TTAGCATAGAGATCCTTCCTG-3´ |
| *Sm22* | 5´-CAACAAGGGTCCATCCTACGG-3´ | 5´-ATCTGGGCGGCCTACATCA-3´ |
| *Klf4* | 5´-GTGCCCCGACTAACCGTTG-3´ | 5´-GTCGTTGAACTCCTCGGTCT-3´ |
| *Mmp9* | 5´-CTGGACAGCCAGACACTAAAG-3´ | 5´-CTCGCGGCAAGTCTTCAGAG-3´ |
| *Cd36* | 5´-GAACCACTGCTTTCAAAAACTGG-3´ | 5´-TGCTGTTCTTTGCCACGTCA-3´ |
| *SRA* | 5´-TGAACGAGAGGATGCTGACTG-3´ | 5´-GGAGGGGCCATTTTTAGTGC-3´ |
| *Abca1* | 5´-AGTGATAATCAAAGTCAAAGGCACAC-3´ | 5´-AGCAACTTGGCACTAGTAACTCTG-3´ |
| *Abcg1* | 5´-TTCATCGTCCTGGGCATCTT-3´ | 5´-CGGATTTTGTATCTGAGGACGAA-3´ |
